# Naphthalene *peri*-Diselenide-Based BODIPY Probe for the Detection of Hydrogen Peroxide, *tert-*Butylhydroperoxide, Hydroxyl Radical, and Peroxynitrite Ion

**DOI:** 10.1021/acsomega.4c05366

**Published:** 2025-02-13

**Authors:** Babli Chhillar, Nikhil Sodhi, Rajni Kadian, Eliane Ribeiro Neres, Manisha Yadav, Manisha Kundu, Vinutha K. Venkatareddy, Rajeswara Rao Malakalapalli, Jamal Rafique, Sumbal Saba, Vijay P. Singh

**Affiliations:** † Department of Chemistry & Centre of Advanced Studies in Chemistry, Panjab University, Sector-14, Chandigarh 160014, India; ‡ LabSO, Instituto de Química – IQ, Universidade Federal de Goiás – UFG, Goiânia 74690-900, GO, Brazil; § Department of Chemistry, Indian Institute of Technology Dharwad, WALMI Campus, Dharwad 580011, Karnataka, India; ∥ Instituto de Química – INQUI, Universidade Federal do Mato Grosso do Sul – UFMS, Campo Grande 79074-460, MS, Brazil

## Abstract

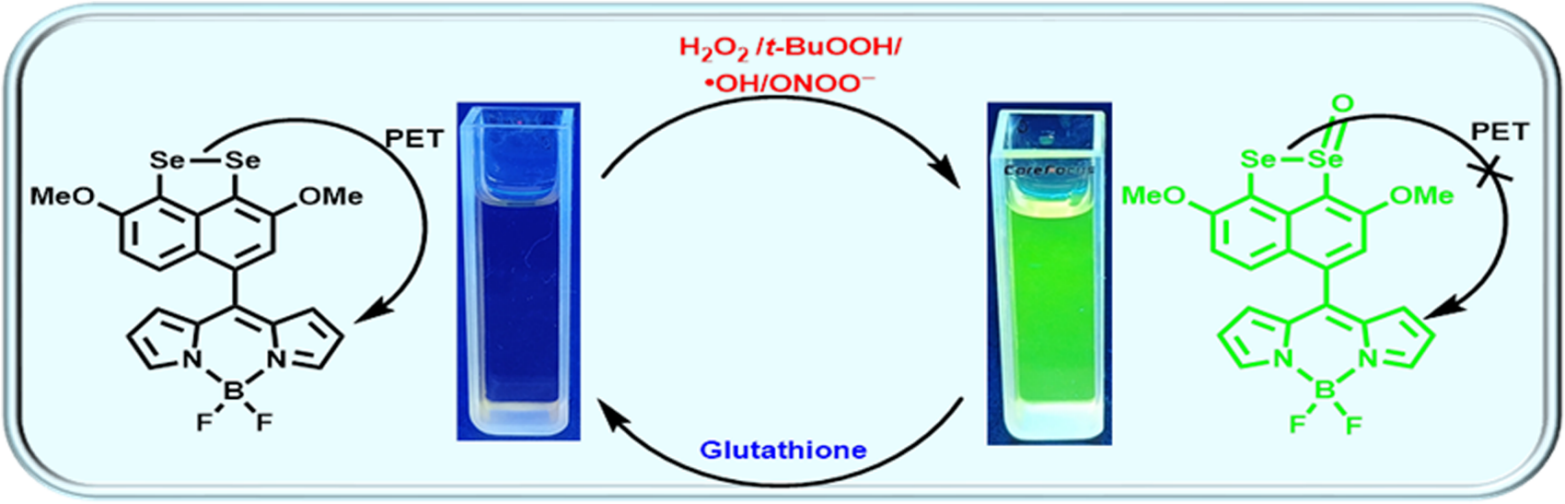

Dimethoxynaphthalene peri-diselenide-based BODIPY (4,4-difluoro-4-bora-3a,4a-diaza-s-indacene) probe has been synthesized. The probe demonstrated selectivity and sensitivity for hydrogen peroxide (H_2_O_2_) and *tert-*butylhydroperoxide (*t-*BuOOH), hydroxyl radical (^•^OH), and peroxynitrite ion (ONOO^–^) detection and reversibility upon treatment with glutathione. The limits of detection of the probe were observed to be 0.40 μM for H_2_O_2_, 0.41 μM for *t*-BuOOH, 0.95 μM for ^•^OH, and 0.46 μM for ONOO^–^, respectively. A proposed mechanism for the “turn-on” event has been suggested and corroborated by spectroscopic and computational data. It has been proposed that electron transfer occurred from the Se center to the BODIPY moiety, followed by the photoinduced electron transfer (PET) mechanism.

## Introduction

Over the last few decades, organoselenium compounds have garnered increasing interest in the field of medicine, enzymology, and bio-organic chemistry.^1^ In particular, these are known to exhibit excellent biological activities including anti-inflammatory, antitumor, antifungal, and antioxidant properties.^2,3^ Selenium is a key component at the active site of crucial glutathione peroxidase (GPx) enzymes.^4^ In the presence of glutathione, it converts biological hydrogen peroxide (H_2_O_2_) into water.^5^ Several organoselenium compounds have been created to replicate the activity of GPx-enzymes.^6−12^ In recent years, selenium,^13,14^ sulfur,^15^ and tellurium-based^16,17^ compounds have appeared as potential sensors for detecting metal ions, reactive nitrogen species (RNS), reactive oxygen species (ROS), and biothiols. The biological system contains ROSs such as superoxide (O_2_^•–^), H_2_O_2_, *tert-*butylhydroperoxide (*t-*BuOOH), hypochlorite (^−^OCl), hydroxide radical (^•^OH), and *tert-*butoxide (*t-*BuO^•^) in conjunction with RNS such as peroxynitrite (ONOO^–^) and nitric oxide (^•^NO).^18^ Each species plays a specific role in cellular processes including signal transduction, neurotransmission, and smooth muscle relaxation. However, the excessive production of these species can disrupt the equilibrium and lead to oxidative stress.^19^ These highly reactive species are associated with various human disorders, including Alzheimer’s disease, Parkinson’s disease, and cancer.^20−22^ Indeed, for monitoring the concentration of ROS, it is crucial to design redox-responsive fluorescent probes with good reversibility.^23^ The application of fluorescence technique in identifying vital biologically important analytes has garnered enormous attention due to its exceptional sensitivity, swift response times, easy implementation, and considerable potential for imaging cells and tissues.^24,25^ Many fluorescent organoselenium probes have been designed for the detection of ROS,^26^ RNS,^27^ and biothiols.^28^ BODIPY (4,4-difluoro-4-bora-3a,4a-diaza-s-indacene) is known for its unique characteristics, such as good photochemical stability, a notable extinction coefficient, and strong fluorescence featuring a high quantum yield.^29−31^

**Figure 1:**
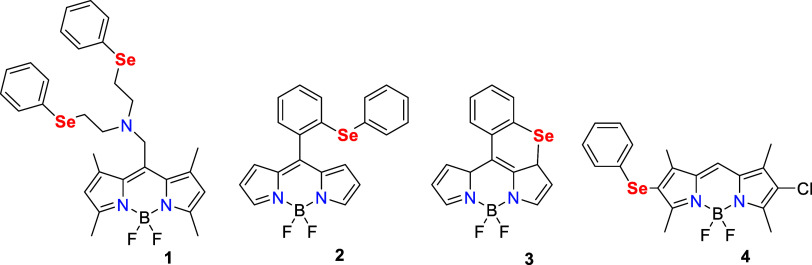
Reported BODIPY-based organoselenium probes **1**–**4**.

Wu et al. prepared a novel BODIPY-based fluorescent organoselenium probe **1** via the reaction of bis[2-(phenylselenyl)ethyl]amine and 8-[chloromethyl]-4,4-difluoro-1,3,5,7-tetramethyl-4-bora-3a,4a-diaza-s-indacene under mild basic conditions in tetrahydrofuran for the detection of metal ions (Figure 1).^32^

The chelation of Cu^2+^ to the chelating groups of probe **1** increased the fluorescence of the BODIPY moiety by inhibiting photoinduced electron transfer (PET) from the Se center to the BODIPY moiety. Another BODIPY-based organoselenium probe **2** was obtained from the oxidation of 2-(phenylselenyl)phenyldipyrromethane with 2,3-dichloro-5,6-dicyano-1,4-benzoquinone (DDQ) subsequently followed by the incorporation of borontrifluoride diethyl etherate (BF_3_·Et_2_O). Probe **2** has shown an intensified fluorescence due to the construction of selenoxide upon the addition of HOCl.^33^ Later, Churchill and co-workers reported a BODIPY-based probe **3**, synthesized from the reaction of bis(*o*-formylphenyl)diselenide with DDQ, BF_3_·Et_2_O, and triethylamine (Et_3_N),^34^ showing a selective detection of ^–^OCl and remarkably quick response with high sensitivity, exhibiting a strong red fluorescence. The observed fluorescence enhancement was attributed to the inhibition of PET from the phenyl selenide group to the BODIPY moiety. The same group has also synthesized a novel “turn-on” phenyl selenide BODIPY-based probe **4** by the reaction of monochlorinated BODIPY compound with phenylselenyl chloride. Probe **4** exhibits high sensitivity and selectivity for ^–^OCl.^35^ Upon the addition of HOCl, probe **4** displayed remarkable fluorescent enhancement by terminating the PET process. Further, Manjare and co-workers developed a Se-containing diBODIPY-based fluorescence probe.^36^ In the presence of O_2_^•–^, the probe showed “turn-on” event due to the quenching of the PET process. Subsequently, a cyclic diselenide-containing BODIPY probe was developed for the sensitive and selective detection of O_2_^•–^.^37^

Organoselenium compounds, when used as small molecular probes for the biosensing of ROSs, allow for the observation of biochemical and biomolecular processes within organisms at the cellular level. Also, organoselenium compounds have a wide range of potential applications, extending from basic biological research to clinical diagnostics. Recently, various organoselenium probes have been reported. Nonetheless, as far as we are aware, there are only a few reports on selenium-based probes for the detection of H_2_O_2_ has been described yet.^38^ Thus, in connection with our continuing interest in organoselenium compounds,^7−12^ we present the synthesis of BODIPY-based molecular probe, having methoxy-substituted cyclic diselenide, used successfully for the selective detection of H_2_O_2_, *t*-BuOOH, ^•^OH, and ONOO^–^.

## Results and Discussion

**Scheme 1:**
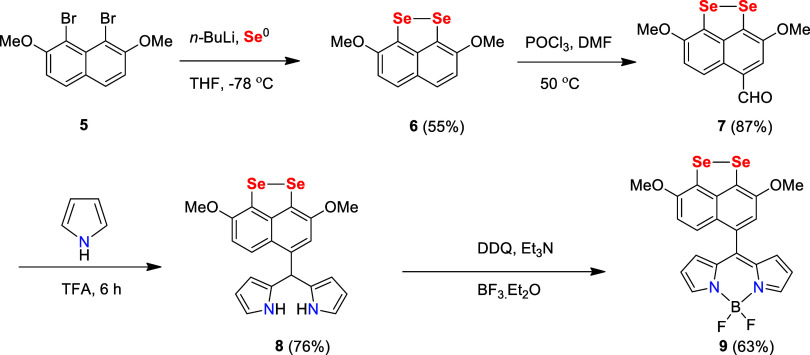
Synthesis of Methoxy-Substituted Cyclic Diselenide-Containing BODIPY-Based Probe **9**

Diselenide **6** was prepared by subjecting 1,8-dibromo-2,7-dimethoxynaphthalene (**5**) to lithium-halogen exchange followed by elemental Se insertion in dry THF at −78 °C (Scheme 1).^39^

Diselenide **6** was fully characterized using the spectroscopic technique, and all data were in good agreement with reported values. During the purification, compound **6** crystallized via slow evaporation in open air, forming purple needle-shaped crystals. Compound **6** was then treated with phosphorus oxychloride (POCl_3_) in dry DMF to yield *p*-formylnaphthyl diselenide **7**. Subsequently, in the presence of catalytic amount of trifluoroacetic acid (TFA), compound **7** was treated with pyrrole (4.5 equiv) under an inert atmosphere to produce dipyrrolemethane diselenide **8**. The desired BODIPY-based probe (methoxy-substituted cyclic diselenide) **9** was prepared from the reaction of compound **8** with DDQ in dichloromethane (DCM) followed by the addition of Et_3_N and BF_3_·Et_2_O. The reaction progress was checked using thin-layer chromatography (TLC), revealing a distinct new spot after each reagent addition. After completion of the reaction, the crude residue was purified by column chromatography and characterized using NMR (^1^H, ^13^C{^1^H}, ^77^Se{^1^H}), and HRMS spectroscopic techniques. For probe **9**, in the ^1^H NMR spectrum, signal for the aliphatic C–H proton disappeared. Additionally, the two signals for two chemically different Se atoms in probe **9** were shifted downfield compared with **8**, further confirming the formation of probe **9** (see Figure S11).

### FTIR Analysis of Probe 9

The FTIR spectrum of probe **9** revealed several key peaks, each associated with specific functional groups (Figure S13). The peak at 3142 cm^–1^ corresponded to the C–H stretching vibration (aromatic rings). The peak at 1545 cm^–1^ is attributed to C=C stretching within the pyrrole ring, reflecting its conjugated double bond system. The 1389 cm^–1^ peak is assigned to C–O stretching in the methoxy (OMe) group, further confirmed by a secondary peak at 1256 cm^–1^. The peak at 1057 cm^–1^ represented the stretching of the C–N bond in the ring structure. The presence of B–N stretching vibrations is indicated by the peak at 782 cm^–1^.^40^ Finally, the 581 cm^–1^ peak is associated with Se–C stretching, indicating a selenium–carbon bond.^41^ This comprehensive spectral profile confirmed the successful incorporation of methoxy, pyrrole, selenium, and boron-containing groups in compound **9**.

### X-ray Crystallographic Structure Analysis

**Figure 2:**
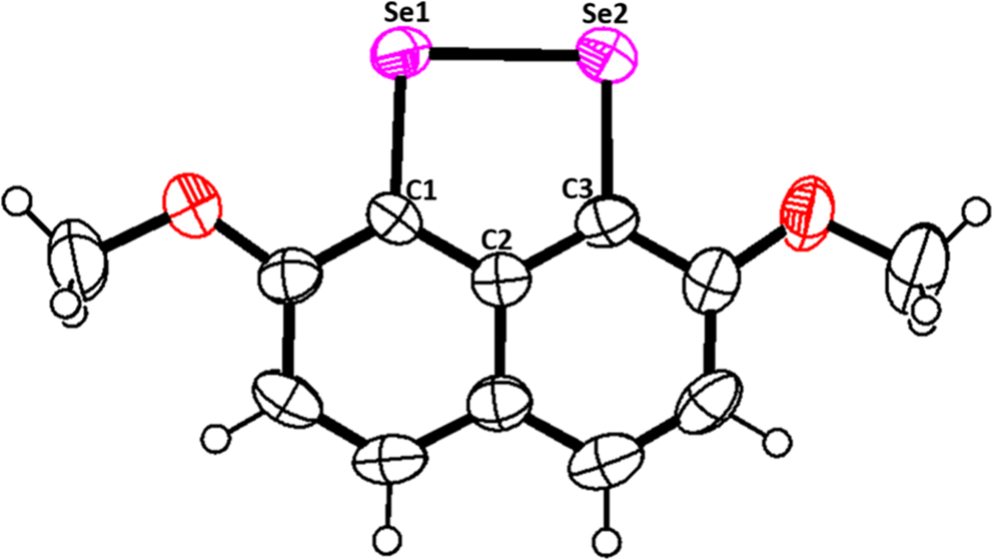
ORTEP view of compound **6** with a 50% probability of thermal ellipsoids. Significant bond lengths [Å] C1–Se1 1.902(6); C3–Se2 1.915(6); Se1–Se2 2.358(11) and angles [deg] C1–Se1–Se2 91.6(2); Se1–Se2–C3 90.8(2); C1–C2–C3 121.1(6); Se1–C1–C2 117.9(5); Se2–C3–C2 118.6(5).

Needle-shaped, purple-colored crystals of compound **6**, suitable for single-crystal X-ray diffraction analysis, were grown through slow evaporation of solvent from a solution of ethyl acetate and *n*-hexane at ambient temperature (Figure 2).

Diselenide **6** was crystallized in monoclinic mode with space group P2_1_/n. The C1–Se1–Se2 and Se1–Se2–C3 bond angles were found to be 90.86 and 91.65, respectively, indicating V-shaped geometry around the Se atoms.

After the successful synthesis of probe **9**, its ability to selectively and sensitively detect biologically important analytes was evaluated. First, the absorption solvatochromic studies were conducted in various solvents ranging from low-polarity hexane to high-polarity methanol, showing minimal effect (see Figure S14). The spectroscopic (UV–vis and fluorescence) properties of probe **9** with ROS were examined in a methanol/water (70:30) mixture. The 70:30 methanol/water mixture was used for the UV–vis and fluorescence studies of probe **9** to provide an optimal solvent environment that was necessary for the probe’s solubility. In the initially taken 50:50 MeOH/water mixture, the probe was not completely soluble. Probe **9** was screened with various ROSs such as ^–^OCl, O_2_^•–^, H_2_O_2_, *t*-BuOOH, *t*-BuO^•^, and ^•^OH in water using a UV–vis spectrophotometer. The results showed an absorption maximum for probe **9** at 500 nm, with a range from 430 to 530 nm (see Figure S15). Probe **9** displayed no emission due to the electron transfer interaction between BODIPY and the selenium moiety. This, electron transfer interaction can be interrupted by the oxidation of selenium to selenoxide which is expected to result in strong emission.

**Figure 3:**
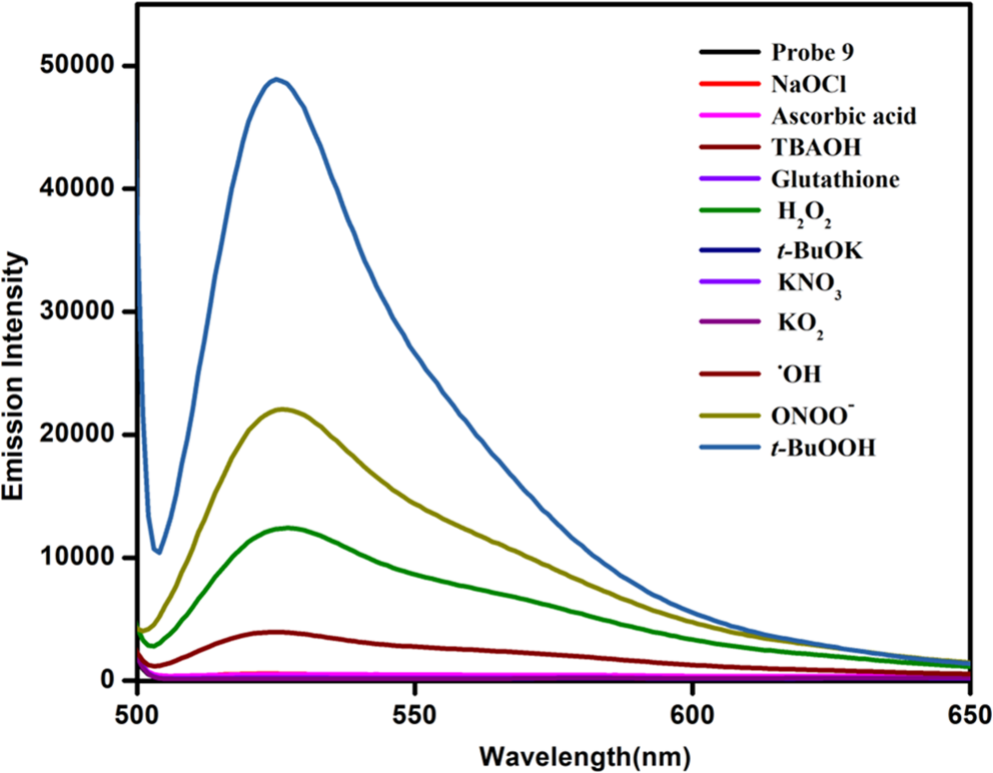
Emission spectra of probe **9** (10 μM, methanol/water: v/v = 70:30) with various ROSs, glutathione, and ascorbic acid (λ_ex_ = 485 nm and λ_em_ = 525 nm).

Therefore, fluorescence studies were carried out for probe **9** with various ROSs/RNSs (^−^OCl, O_2_^•–^, H_2_O_2_, *t*-BuOOH, *t*-BuO^•^, ^•^OH, and ONOO^–^), glutathione, and ascorbic acid. Probe **9** displayed absorption at 485 nm and emission at 525 nm, exhibiting almost negligible emission intensity (Figure 3).

Upon addition of H_2_O_2_, probe **9** displayed strong yellow “turn-on” fluorescence over the other ROSs/RNSs (^−^OH, ^–^OCl, O_2_^•–^, and NO_3_^–^) (Figure 3), glutathione, and ascorbic acid due to the oxidation of selenium to selenoxide (see Figure S16). It also showed some shift in the presence of *t*-BuOOH, ^•^OH, and ONOO^–^. However, relatively high reactivity was observed toward H_2_O_2_ (Figure 3).

**Figure 4:**
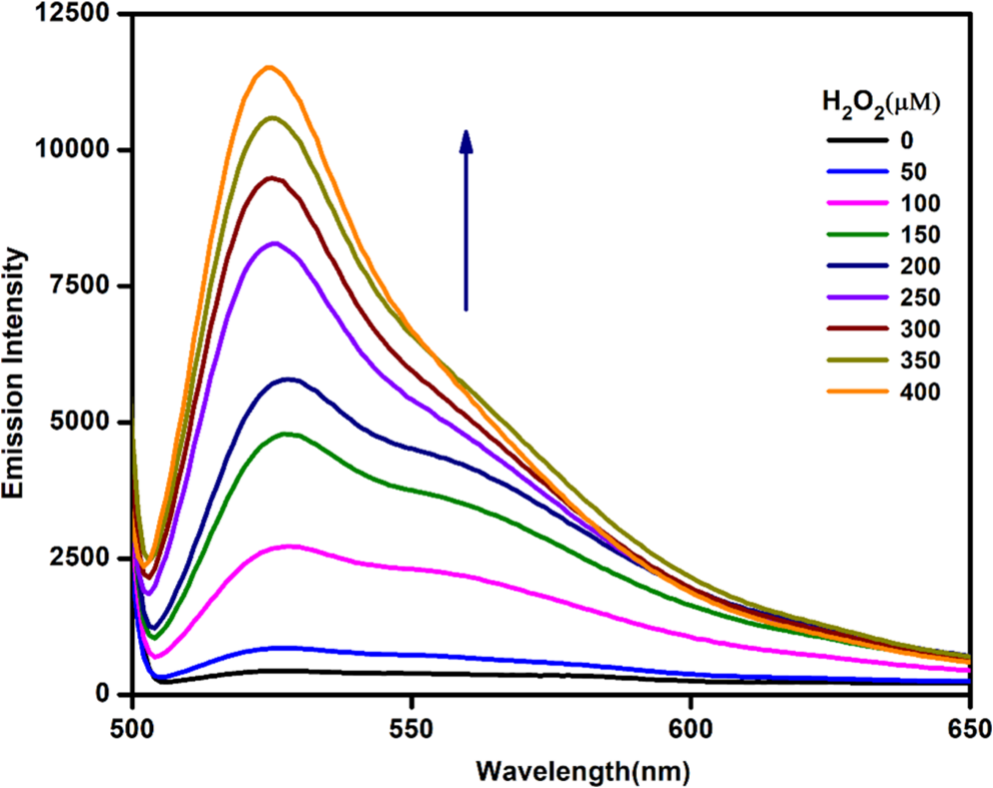
Emission spectra of **9** (10 μM, methanol/water: v/v = 70:30) with increasing concentrations of H_2_O_2_ (0–400 μM) (λ_ex_ = 485 nm and λ_em_ = 525 nm).

By increasing the concentration study, a linear increase in the fluorescence intensity was noticed with a gradual increase in H_2_O_2_ concentrations from 0 to 400 μM (Figure 4).

**Figure 5:**
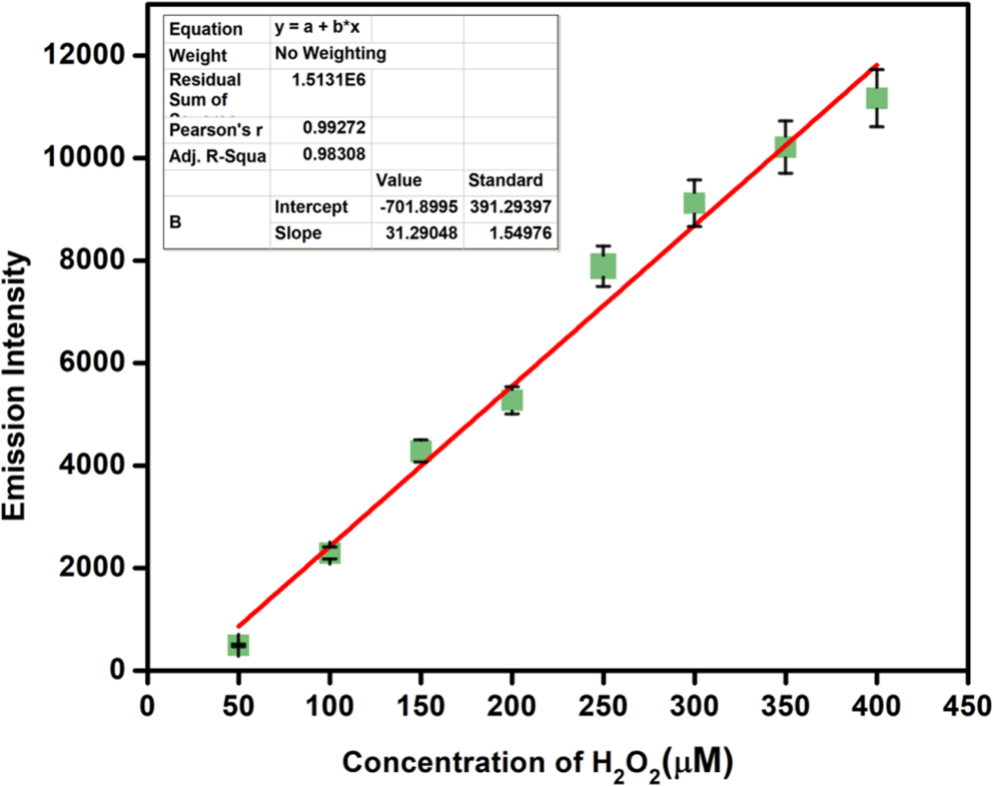
Plot for the calculation of LOD from the emission of **9** (10 μM, methanol/water: v/v = 70:30) with increasing concentrations of H_2_O_2_ (0–400 μM) (λ_ex_ = 485 nm and λ_em_ = 525 nm).

Probe **9** also showed a linear increase in fluorescence intensity upon a gradual increase in the concentration of *t*-BuOOH, ^•^OH, and ONOO^–^ (see Figures S17–S19). The limit of detection (LOD) of the probe was established by plotting the intensity against concentrations of H_2_O_2_, *t*-BuOOH, ^•^OH, and ONOO^–^ (Figures 5 and S20–S22, respectively). The LOD values of probe **9** were found to be 0.40 μM for H_2_O_2_, 0.41 μM for *t*-BuOOH, 0.95 μM for ^•^OH and 0.46 μM for ONOO^–^, respectively.

**Figure 6:**
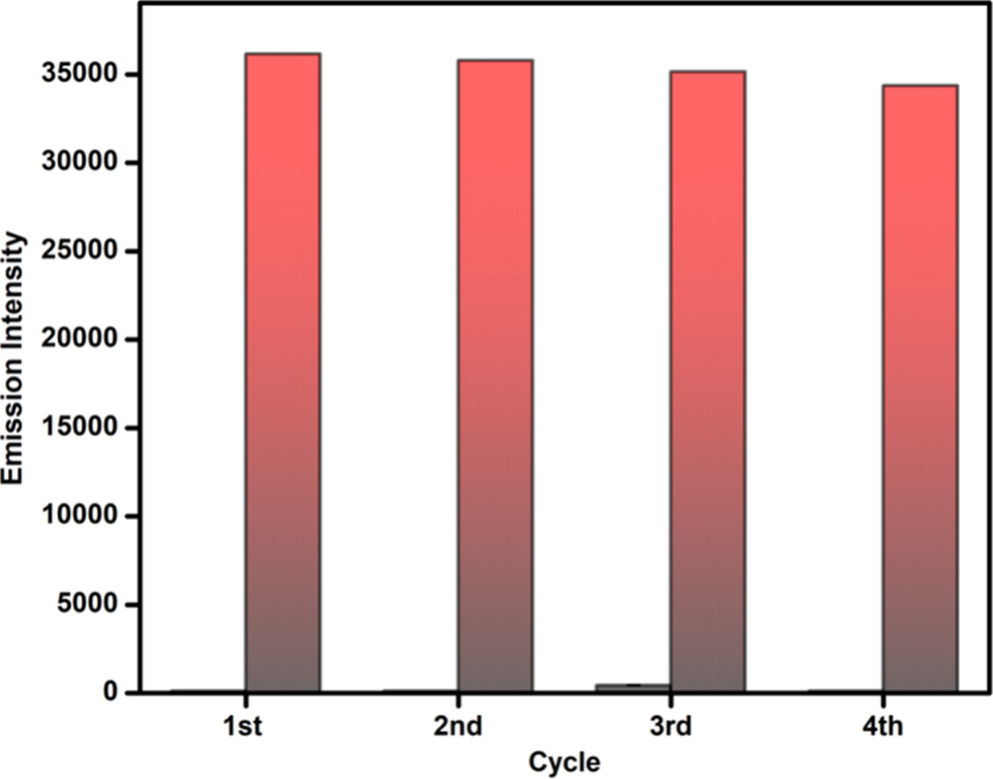
Fluorescence response before and after the addition of glutathione to the solution of probe **9** (10 μM, methanol/water: v/v = 70:30) and H_2_O_2_ (λ_ex_ = 485 nm and λ_em_ = 525 nm).

To assess the redox capacity of Se in probe **9**, the probe was initially oxidized with H_2_O_2_ and subsequently treated with biothiol glutathione. A significant decrease in the fluorescence emission intensity was observed, affirming the reversibility of probe **9**. The reduced probe was then further oxidized with H_2_O_2_ and a significant increase in fluorescent intensity was observed (Figure 6, cycle first). This process was analyzed for four cycles (cycles 1st–4th) for the alternate addition of H_2_O_2_ and glutathione.

**Figure 7:**
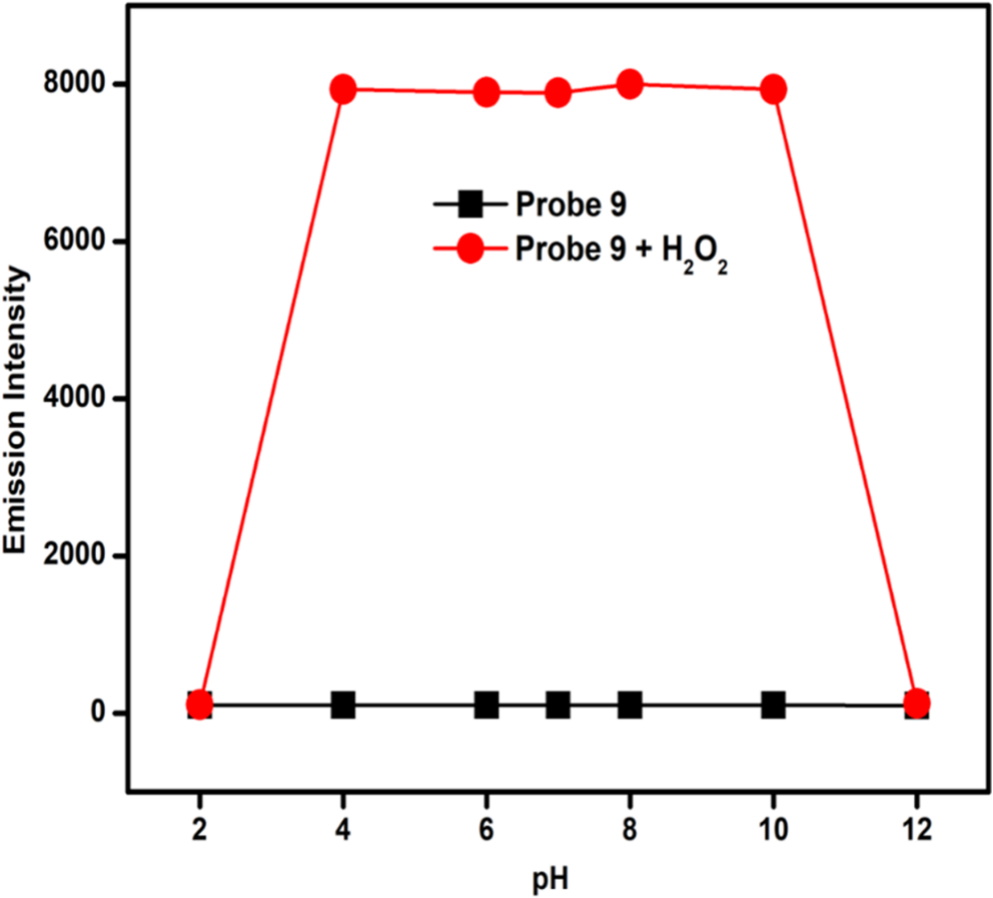
Fluorescence intensity measurements on probe **9** (10 μM) and the probe in the presence of 300 μM H_2_O_2_ under different pH ranges, λ_ex_ = 485 nm and λ_em_ = 525 nm.

A pH-dependent experiment was performed with probe **9** to determine the optimal range for H_2_O_2_ sensing. The emission intensities of probe **9** are significantly low within the pH range of 2–12 (Figure 7). Upon the addition of H_2_O_2_, a notable increase in emission intensity at 525 nm was observed within a pH range of 4–10, which means that the probe could be used to detect H_2_O_2_ at the pH range of 4–10. At pH 12, the probe in the presence of H_2_O_2_ remains nonfluorescent.

**Figure 8:**
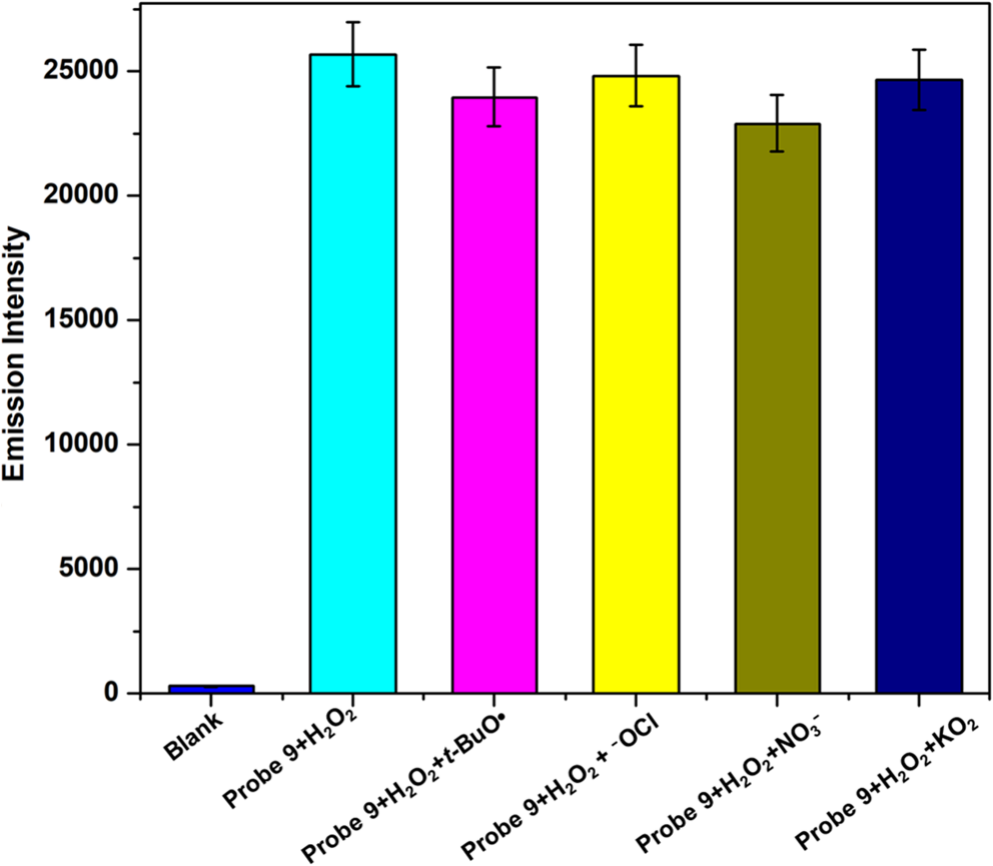
Fluorescence spectra of probe **9** (10 μM, methanol/water 70:30) and H_2_O_2_ with other ROS (*t-*BuOK, NO_3_^–^, ^–^OCl, KO_2_) (λ_ex_ = 485 nm and λ_em_ = 525 nm).

An interference study was conducted for probe **9** in the presence of H_2_O_2_ and various other ROSs (*t-*BuOK, NO_3_^–^, ^–^OCl, and KO_2_) in Figure 8. The interferents such as *t-*BuOK, NO_3_^–^, OCl^–^, and KO_2_ have been included based on their potential to interact with the target probe **9**, potentially affecting the accuracy and reliability of the results. No significant change in the fluorescence intensity of the probe with H_2_O_2_ was observed in the presence of other ROSs. These results indicate that other ROSs do not interfere with the detection of H_2_O_2_ by probe **9**.

**Figure 9:**
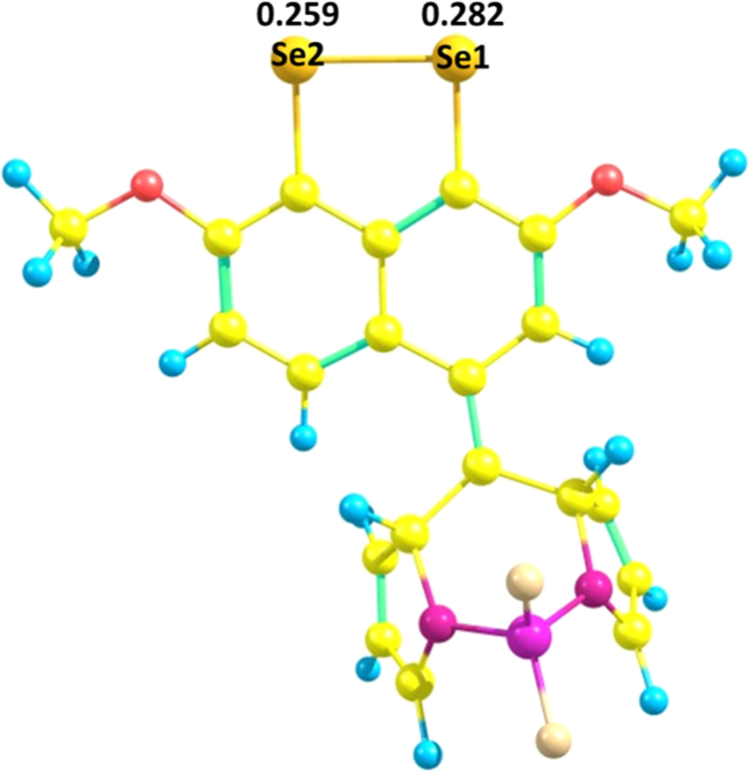
Optimized geometry of probe **9**.

Density functional theory (DFT) calculations were carried out to provide theoretical support for the experimentally observed “turn-on” fluorescence response during the detection of H_2_O_2_ and *t-*BuOOH. The geometry of probe **9** was fully optimized at the B3LYP/6-311+G(d,p) level of theory in the gas phase as shown in Figure 9 (see Table S8).

Natural bond orbital (NBO) analysis at the B3LYP/6-311++G(d,p) level was used to investigate the charge density on the Se atoms, employing the B3LYP/6-311+G(d,p) level-optimized geometry. The analysis indicated that Se1 and Se2 exhibited positive charge densities of 0.282 and 0.259, respectively (Figure S23). Additionally, in the ^77^Se{^1^H} NMR spectrum of probe **9**, two signals at 426 and 418 ppm were observed, corresponding to Se1 and Se2, respectively. The analysis indicated that Se1 was more electron deficient than Se2. Based on the observed higher positive charge density on Se1, it was predicted that nucleophilic attack by H_2_O_2_/*t-*BuOOH would occur preferentially at this site.

**Scheme 2:**
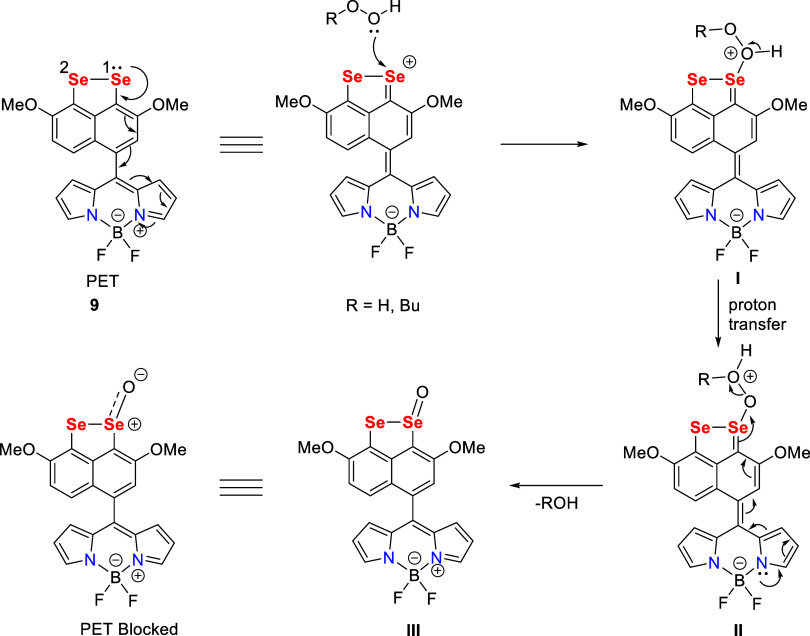
Proposed Mechanism for the “Turn-on” Response of Probe **9** with H_2_O_2_ and *t-*BuOOH

A plausible mechanism for a “turn-on” event for probe **9** has been proposed as shown in Scheme 2.

In the proposed mechanism, electron transfer from the Se center to the BODIPY moiety resulted in PET quenching. As suggested from NBO analysis, the nucleophile H_2_O_2_/*t-*BuOOH attacked on the electron-deficient Se1 center. Further, intramolecular proton transfer would take place within intermediate **I** to produce intermediate **II**. Intermediate **II** rearranged to generate selenoxide **III** along with the elimination of the water/butyl alcohol molecule. The formation of selenoxide intermediate **III** was confirmed by the mass spectrum analysis of a mixture of probe **9** with H_2_O_2_. The isotopic pattern of Se with monooxidation was observed (see Figure S24). It was concluded that Se1(II) would oxidize to Se1(IV) and there was no electron density for transfer from the Se center to the BODIPY moiety. Thus, the PET mechanism was blocked and significant enhancement in fluorescence intensity was observed. The PET mechanism is well documented in previous research studies.^33,42^

**Figure 10:**
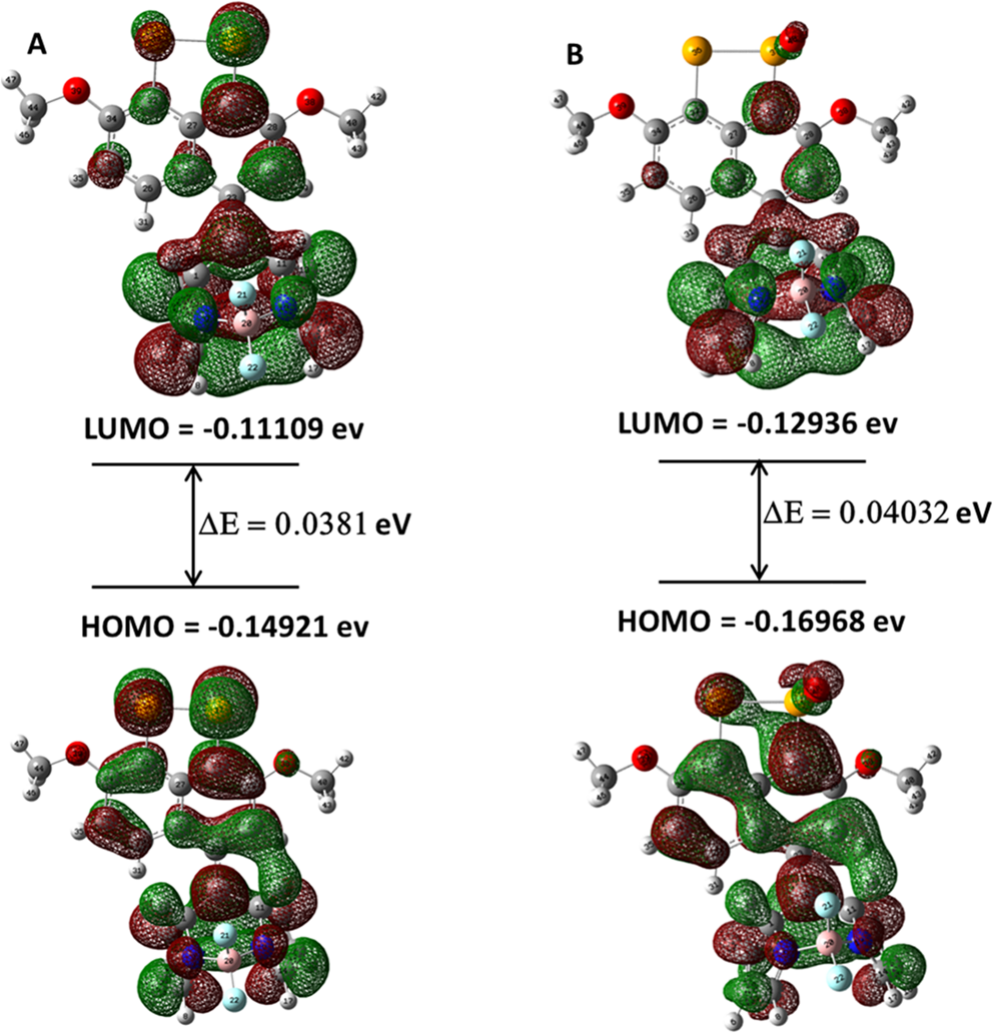
HOMO and LUMO of (A) probe **9**; (B) selenoxide **III** in gas phase calculations (B3LYP/6-311G+(d,p)).

To provide further evidence for the occurrence of PET quenching, the frontier molecular orbitals (FMOs) of probe **9** and its oxidized form, selenoxide **III**, were investigated and are depicted in Figure 10 (see also Figures S25 and S26). The geometry of selenoxide **III** was fully optimized at the B3LYP/6-311+G(d,p) level of theory (see Table S8).

Analysis of the frontier molecular orbitals revealed that the highest occupied molecular orbital (HOMO) of probe 9 was primarily localized on the naphthalene core, whereas the lowest unoccupied molecular orbital (LUMO) was predominantly localized on the BODIPY core (Figure 10A). Electron transfer from HOMO to LUMO of probe **9** corresponded to its quenched state.^32^ However, in selenoxide **III**, sustained electron density was observed within both HOMO and LUMO of the BODIPY core (Figure 10B). Consequently, this alteration led to a significant enhancement in the fluorescence intensity exhibited by probe **9**.

## Conclusions

In summary, probe **9** was developed for the selective and sensitive detection of H_2_O_2_ and *t-*BuOOH, ^•^OH, and ONOO^–^. H_2_O_2_, *t-*BuOOH, ^•^OH, and ONOO^–^ were the species that showed a significant increase in fluorescence intensity in the absorption spectra. The probe’s oxidized form was reversibly generated with glutathione. The “turn-on” mechanism was proposed and supported with the DFT calculations. This study aims to investigate the potential application of a small organoselenium molecular probe for the detection of H_2_O_2_, *t-*BuOOH, ^•^OH, and ONOO^–^ in living organisms.

## Experimental Section

All reactions were monitored by TLC using a UV lamp (λ = 254 nm). ^1^H and ^13^C{^1^H} NMR spectra for all synthesized products were recorded on 400 MHz (^1^H: 400 MHz, ^13^C{^1^H}: 100 MHz) and 500 MHz (^1^H: 500 MHz, ^13^C{^1^H}: 125 MHz) spectrometer. Solvent residual peaks were used as an indirect reference to tetramethylsilane (TMS) (δ = 0 ppm), i.e., CDCl_3_ (^1^H: δ = 7.26; ^13^C: δ = 77.2 ppm) and DMSO-d_6_ (^1^H: δ = 2.50; ^13^C: δ = 39.5 ppm). The ^77^Se{^1^H} NMR spectra were obtained on a 500 MHz (^77^Se{^1^H}: 100 MHz) spectrometer, using Ph_2_Se_2_ (δ = 463 ppm) as an indirect reference to Me_2_Se (δ = 0 ppm). For purification, silica gel (60–120 mesh) size was utilized in flash column chromatography. The melting points for all compounds were determined without corrections using a digital melting point apparatus. HRMS spectra were acquired on a time-of-flight (TOF) mass spectrometer with electrospray ionization (ESI) in positive ion mode. A Cary 5000 UV–vis–NIR spectrophotometer was employed for absorption measurements, and fluorescence spectra were recorded on a PerkinElmer FL6500 spectrophotometer.

### Synthesis of 2,7-Dimethoxynaphtho[1,8-cd]-1,2-dislenole (**6**)^39^

*n-*Butyllithium (4 mL, 2.0 M, 8 mmol) was added to a solution of 1,8-dibromo-2,7-dimethoxynaphthalene (1.0 g, 2.88 mmol) in dry THF at −78 °C under an inert atmosphere (N_2_). After 1.5 h of the reaction, elemental Se was introduced at 0 °C and the reaction mixture was further allowed to stir overnight at room temperature. The solution was quenched with ammonium chloride and stirred for 0.5 h in open air. The organic layer was extracted with dichloromethane (DCM, 3 × 20 mL) and washed with brine. The combined organic layers were dried over anhydrous sodium sulfate (Na_2_SO_4_) and concentrated under reduced pressure. Purification was performed using column chromatography and ethyl acetate/*n-*hexane (4%) as an eluent, affording diselenide **6**. Yield: 0.45 g (55%), mp 150–151 °C (mp 155–156 °C, literature); purple solid. ^1^H NMR (400 MHz, CDCl_3_) δ 7.60 (s, 1H), 6.98 (d, *J* = 8.72 Hz, 1H), 4.49 (d, *J* = 18.8 Hz, 3H) 1.64 (s, 3H).

### Synthesis of *p*-Formylnaphthyl Diselenide **7**

Diselenide **6** (0.3 g, 0.87 mmol) was dissolved in dry DCM and POCl_3_ (0.23 g, 0.14 mL, 1.5 mmol) was added dropwise at 0 °C. The reaction mixture was then heated at 50 °C for overnight. After the completion of the reaction, it was quenched with water and extracted with ethyl acetate (3 × 20 mL), washed with brine solution, dried over anhydrous Na_2_SO_4_, and concentrated under reduced pressure, resulting in a red-colored residue. Purification was performed using column chromatography and ethyl acetate/*n-*hexane (4%) as an eluent. Orange-colored compound **7** was obtained as a product. Yield: 0.28 g (87%), m.p.: 214–216 °C. ^1^H NMR (500 MHz, CDCl_3_) δ 10.25 (s, 1H), 8.97 (d, *J* = 10.0 Hz, 1H), 7.41 (s, 1H), 7.17 (d, *J* = 10.0 Hz, 1H), 4.06 (s, 3H), 4.03 (s, 3H).^13^C{^1^H} NMR (125 MHz, CDCl_3_) δ 191.0, 153.7, 152.2, 140.9, 136.3, 129.9, 125.4, 124.2, 123.2, 119.9, 113.5, 56.6, 56.5. ^77^Se{^1^H} NMR (100 MHz, CDCl_3_) δ 448, 429. HRMS (ESI/TOF) *m*/*z* [M]^+^ calcd. for C_13_H_10_O_3_Se_2_, 373.8960, found 373.8943.

### Synthesis of Dipyrrolemethane Diselenide **8**

To the solution of diselenide **7** (0.35 g, 0.94 mmol), pyrrole (3 mL) and a catalytic amount of TFA (0.05 mL) were added. The reaction mixture was allowed to stir for 6 h at room temperature. The progress of the reaction was monitored by TLC. After completion of the reaction, it was quenched with water and extracted with DCM (3 × 20 mL). The combined organic layers were dried over anhydrous Na_2_SO_4_ and evaporated to dryness under reduced pressure using a rotary evaporator. Purification was performed using column chromatography and ethyl acetate/*n-*hexane (10%) as an eluent, affording purple color compound **8** as a product. Yield: 0.34 g (76%), m.p.: >300 °C. ^1^H NMR (500 MHz, DMSO-*d*_6_) δ 7.83 (d, *J* = 5.0 Hz, 1H), 7.15 (d, *J* = 10.0 Hz, 1H), 6.74 (s, 1H), 6.62 (s, 2H), 5.96 (s, 1H), 5.90 (m, 2H), 5.67 (s, 2H), 3.93 (s, 3H), 3.74 (s, 3H). ^13^C{^1^H} NMR (125 MHz, DMSO-*d*_6_) δ: 153.1, 152.6, 140.2, 139.6, 132.9, 126.1, 123.2, 122.5, 120.5, 117.4, 112.4, 112.3, 107.4, 107.0, 56.8, 56.5. ^77^Se{^1^H} NMR (100 MHz, DMSO-*d*_6_) δ 407, 398. HRMS (ESI/TOF) *m*/*z* [M]^+^ calcd. for C_21_H_18_N_2_O_2_Se_2_, 489.9699, found 489.9690.

### Synthesis of Cyclic Diselenide-BODIPY Probe **9**

DDQ (0.33 g, 1.43 mmol) was added to the solution of dipyrrolemethane diselenide **8** (0.35 g, 0.72 mmol) in DCM (20 mL), and the mixture was stirred at RT for 45 min under nitrogen atmosphere. NEt_3_ (2.06 mL, 14.75 mmol) was added to the mixture and the reaction mixture was allowed to stir for 10 min, followed by the addition of 14.75 mmol BF_3_·Et_2_O (1.82 mL) and the reaction was left to stir for 3 h at room temperature. The progress of the reaction was monitored by TLC. After completion of the reaction, the reaction was quenched with water and extracted with DCM (3 × 20 mL). The combined organic layers were dried over anhydrous Na_2_SO_4_ and evaporated to dryness under reduced pressure using a rotary evaporator. Purification was performed using column chromatography and ethyl acetate/*n-*hexane (6–10%) as an eluent, affording green-colored solid compound **8** as a product. Yield: 0.24 g (63%), m.p.: 234–236 °C; IR (KBr) 3142, 1545,1389, 1256, 782, 581 cm^–1^. ^1^H NMR (500 MHz, DMSO-*d*_6_) δ 8.15 (s, 2H), 7.35 (d, *J* = 6.5 Hz, 2H), 7.09 (d, *J* = 9.5 Hz, 1H), 6.83 (d, *J* = 3.5 Hz, 2H), 6.57 (d, *J* = 2.5 Hz, 2H), 3.95 (s, 3H), 3.89 (s, 3H). ^13^C{^1^H} NMR (125 MHz, DMSO-*d*_6_) δ: 152.8, 151.7, 145.0, 144.5, 139.5, 135.0, 131.1, 128.3, 126.3, 126.1, 123.5, 122.3, 119.2, 114.6, 112.6, 56.7, 56.3. ^77^Se{^1^H} NMR (100 MHz, DMSO-*d*_6_) δ 426, 418. HRMS (ESI/TOF) *m*/*z* [M]^+^ calcd. for C_21_H_15_BF_2_N_2_O_2_Se_2_, 535.9525, found 535.9513. FTIR (KBr) 3142, 1545, 1389, 1256, 1057, 782, 581 cm^–1^.

### X-ray Crystallographic Study

X-ray crystallographic study of compound **6** was performed using graphite-monochromatized Mo–Kα radiation (λ = 0.71073 Å). The single crystal of compound **6** was mounted on a SuperNova, Single source at offset/far, HyPix3000 diffractometer. Data collection was carried out at 293(2) K. The structure of the compound was solved using Olex2^43^ and refined through a full-matrix least-squares procedure on *F*^2^ for all reflections with SHELXL-2016 software.^44^ Hydrogen atoms were positioned by geometrical means and refined using a riding model. Their isotropic thermal parameters were set at 1.5 times *U*(eq) of the corresponding carbon atoms for sp^3^ C–H bonds and 1.2 times *U*(eq) of the corresponding carbon atoms for sp^2^ C–H bonds. The crystallographic data associated with the structure presented in this study have been archived with the Cambridge Crystallographic Data Centre (CCDC) under accession number CCDC 2322340 (compound **6**). These data can be obtained free of charge from CCDC. Data for compound **6**: C_24_H_20_O_4_Se_4_, *M*_r_ = 688.24, monoclinic, space group *P*2_1_/n, *a* = 11.9484(3) Å, *b* = 12.3700(4) Å, *c* = 16.1224(4) Å, α = 90°, β = 102.636(3)°, γ = 90°, *V* = 2325.20(11) Å^3^, *Z* = 4, *T* = 293(2) K, ρ_(calcd)_ = 1.966 g/cm^3^, GOF = 1.067, *R*_1_ = 0.0742, *w*R**_2_ = 0.1196 (*I* > 2σ (*I*)); *R*_1_ = 0.1403, *w*R**_2_ = 0.1367 (all data). Of the 30,471 reflections that were measured, 5009 were unique (*R*_int_ = 0.0909).

### Photophysical Study

#### Details of UV–Vis and Fluorescence Measurements

The emission of probe **9** was measured using spectrophotometric titration in a mixture of methanol and water. The stock solution of probe **9** (10 μM) was prepared in methanol/water (v/v = 70:30; 10 μM). ROSs/RNSs (^−^OCl, O_2_^•–^, H_2_O_2_, *t*-BuOOH, *t*-BuO^•^, ^•^OH, ONOO^–^) were prepared at a concentration of 10 mM in distilled water. A 5 mL sample of probe **9** (10 μM) was mixed with these ROSs, and the probe’s emission was subsequently measured. The λ_max_ absorption for probe **9** was 500 nm and probe **9** was excited at λ_max_ emission of 525 nm. The graphs were plotted using Origin 2019b.

#### Evaluation of Probe **9** for ROSs: Screening and Sensitivity Studies

Probe **9** was subjected to the screen with different ROSs/RNSs, including ^–^OCl, O_2_^•–^, H_2_O_2_, *t*-BuOOH, *t*-BuO^•^, ^•^OH, ONOO^–^, glutathione, and ascorbic acid. For this experimental procedure, 5 mL of probe **9** at a concentration of 10 μM was combined with ROSs in water. The photometric titration experiment involved gradually increasing the concentration of H_2_O_2_, *t*-BuOOH, ^•^OH, and ONOO^–^ (ranging from 0 to 400 μM). UV–vis and fluorescence measurements were subsequently performed after mixing.

#### Determination of the Detection Limit

The determination of the LOD values was carried out by analyzing the fluorescence concentration curve. The fluorescence emission spectrum of probe **9** was measured three times, and the standard deviation of the seven blank measurements was calculated. To determine the slope, the fluorescence intensity at a specific wavelength of 525 nm was plotted against the concentrations of H_2_O_2_, *t*-BuOOH, ^•^OH, and ONOO^–^. The limit of detection was determined using the following equation:
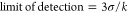
where σ represents the standard deviation of seven blank measurements and *k* is the slope relating fluorescence intensity to the concentrations of H_2_O_2_, *t-*BuOOH, ^•^OH, and ONOO^–^.

### Computational Details

All geometrical calculations were performed using the Gaussian 09 suite of quantum chemistry software.^45^ Density functional theory (DFT) calculations were conducted using the hybrid Becke 3 Lee–Yang–Parr (B3LYP) exchange-correlation functional.^46^ The geometry optimization was carried out with the B3LYP/6-311+G(d,p) basis sets. The charge on the Se atoms was evaluated by natural bond orbital (NBO) analysis at the B3LYP/6-311++G(d,p) level.^47^

## Data Availability

The data that support the findings of this study are available in the Supporting Information.

## Supporting Information Available

^1^H, ^13^C{^1^H}, ^77^Se{^1^H} NMR, and HRMS spectra for all new compounds; UV–vis and fluorescence spectra (PDF)
