# Supplementary material for: Naphthalene peri-Diselenide-Based BODIPY Probe for the Detection of Hydrogen Peroxide, tert-Butylhydroperoxide, Hydroxyl Radical, and Peroxynitrite Ion
Source: ACS Omega. 2025 Feb 13;10(7):6396–405. doi: 10.1021/acsomega.4c05366 (PMC11865972; doi:10.1021/acsomega.4c05366)

Supporting Information

for

Naphthalene *peri*-Diselenide based BODIPY Probe for the detection of Hydrogen Peroxide *tert*-Butylhyroperoxide, Hydroxyl Radical and Peroxynitrite Ion

Babli Chhillar,[a] Nikhil Sodhi,[a] Rajni Kadian,[a] Eliane Ribeiro Neres,[b] Manisha Yadav,[a] Manisha Kundu,[a] Vinutha K. Venkatareddy,[c] Rajeswara Rao Malakalapalli[c] Jamal Rafique,*[b],[d] Sumbal Saba,*[b] and Vijay P. Singh*[a],[d]

[a] Department of Chemistry & Centre of Advanced Studies in Chemistry, Panjab University, Sector-14, Chandigarh – 160 014, India

[b] LabSO, Instituto de Química – IQ, Universidade Federal de Goiás – UFG, Goiânia 74690-900, GO, Brazil

[c] Department of Chemistry, Indian Institute of Technology Dharwad, WALMI Campus, Karnataka – 580 011, India

[d] Instituto de Química – INQUI, Universidade Federal do Mato Grosso do Sul – UFMS, Campo Grande, 79074-460, MS, Brazil

*Email: jamal.rafique@ufms.br (J.R.); sumbalsaba@ufg.br (S.S.); vijay@pu.ac.in (V.P.S.)

---Table of Contents---

p S4-S15 **Figure S1-S12.** ^{1}H, $^{13}C\{^{1}H\}$, $^{77}Se\{^{1}H\}$ NMR and HRMS spectra of synthesized compounds **7-9**.

p S16 **Figure S13**. FTIR analysis of probe **9**

p S17-S22 **Table S1-S7.** X-ray crystallographic data of compound **6.**

p S22 **Figure S14.** The absorption solvatochromic studies of probe **9** (10 µM) with various solvents

p S23 **Figure S15.** UV-Vis spectral changes of probe **9** (10 µM, methanol/water: v/v = 70:30) with various ROSs (1.20 M)

p S23 **Figure S16.** Digital photos of probes **9**, probes **9** + H_2O_2, probes **9** + *t*-BuOOH and probes **9** + other ROS (1.0 M) taken under (A) natural light and (B) UV light

p S24 **Figure S17.** The emission spectra of **9** (10 µM, methanol/water: v/v = 70:30) with increasing concentrations of *t*-BuOOH (0–300 µM); (λ_{ex} = 485 nm and λ_{em} = 525 nm).

p S24 **Figure S18.** Emission of **9** (10 µM, methanol/water: v/v = 70:30) with increasing concentrations of $^{\bullet}OH$ (0–300 µM); (λ_{ex} = 485 nm and λ_{em} = 525 nm).

p S25 **Figure S19.** Emission of **9** (10 µM, methanol/water: v/v = 70:30) with increasing concentrations of $ONOO^{-}$ (0–300 µM); (λ_{ex} = 485 nm and λ_{em} = 525 nm).

p S25 **Figure S20.** Plot for the calculation of LOD from the emission of **9** (10 µM, methanol/water: v/v = 70:30) with increasing concentrations of *t*-BuOOH (0–300 µM); (λ_{ex} = 485 nm and λ_{em} = 525 nm).

p S26 **Figure S21.** Plot for the calculation of LOD from the emission of **9** (10 µM, methanol/water: v/v = 70:30) with increasing concentrations of $^{\bullet}OH$ (0–300 µM); (λ_{ex} = 485 nm and λ_{em} = 525 nm).

p S26 **Figure S22.** Plot for the calculation of LOD from the emission of **9** (10 µM, methanol/water: v/v = 70:30) with increasing concentrations of $ONOO^-$ (0–200 µM); (λ_{ex} = 485 nm and λ_{em} = 525 nm).

p S27 **Table S8.** Optimized geometries and Cartesian coordinates of probe **9** and selenoxide **III**

p S28 **Figure S23.** NBO charge density diagram of probe **9** at B3LYP/6-311++G(d,p) level on the B3LYP/6-311+G(d,p) level-optimized geometry.

p S29 **Figure S24.** Expansion of mass spectrum of mixture of probe **9** and H_2O_2

p S30 **Figure S25.** HOMO-LUMO diagram of probe **9** using the B3LYP/6-311+G(d,p) level-optimized geometry.

p S31 **Figure S26.** HOMO-LUMO diagram of selenoxide **III** using the B3LYP/6-311+G(d,p) level-optimized geometry.

Figure S1. ^{1}H NMR spectrum of compound **7** in $CDCl_3$

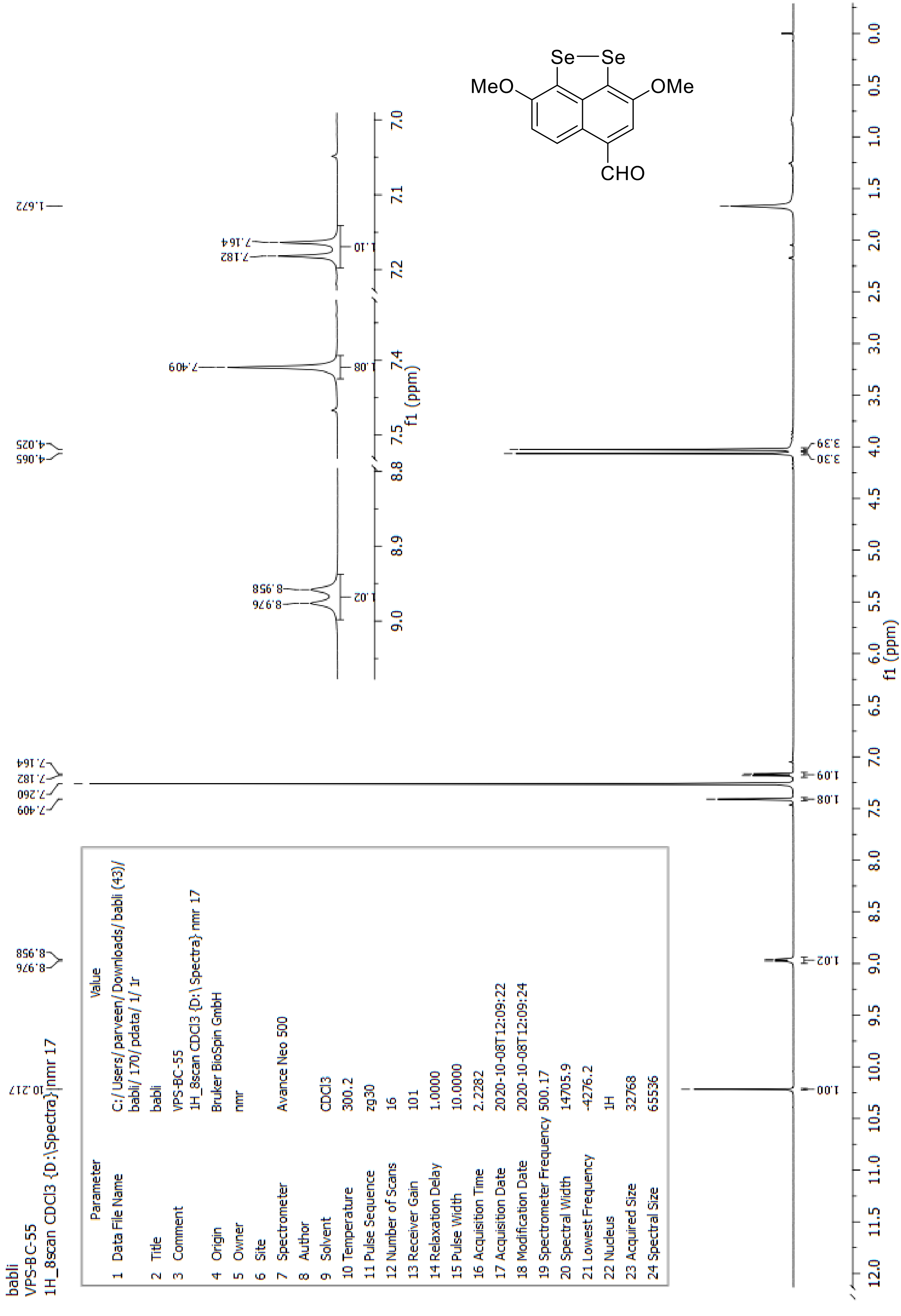

Figure S2. $^{13}C\{^{1}H\}$ NMR spectrum of compound **7** in $CDCl_3$

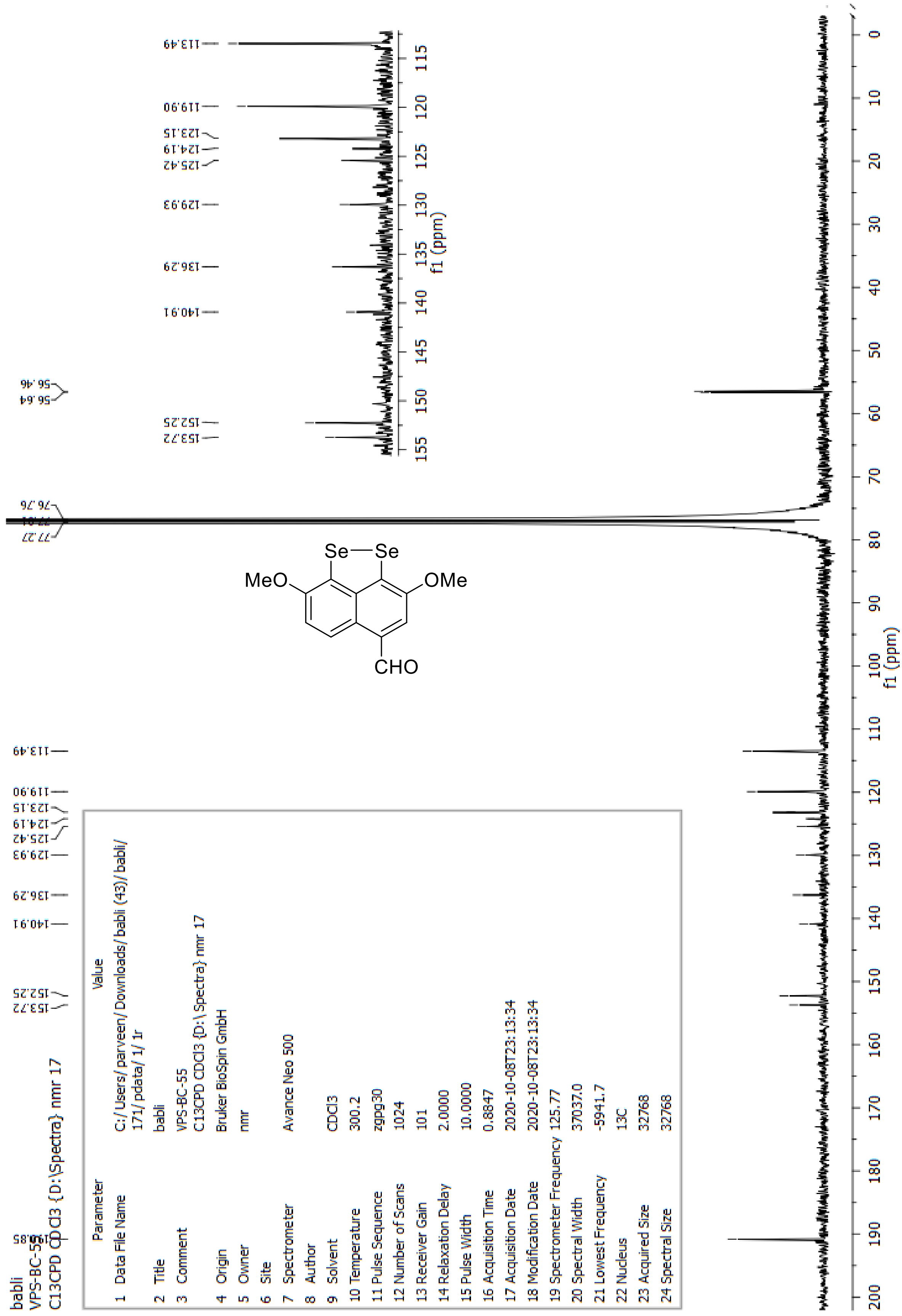

Figure S3. $^{77}Se\{^1H\}$ NMR spectrum of compound **7** in $CDCl_3$

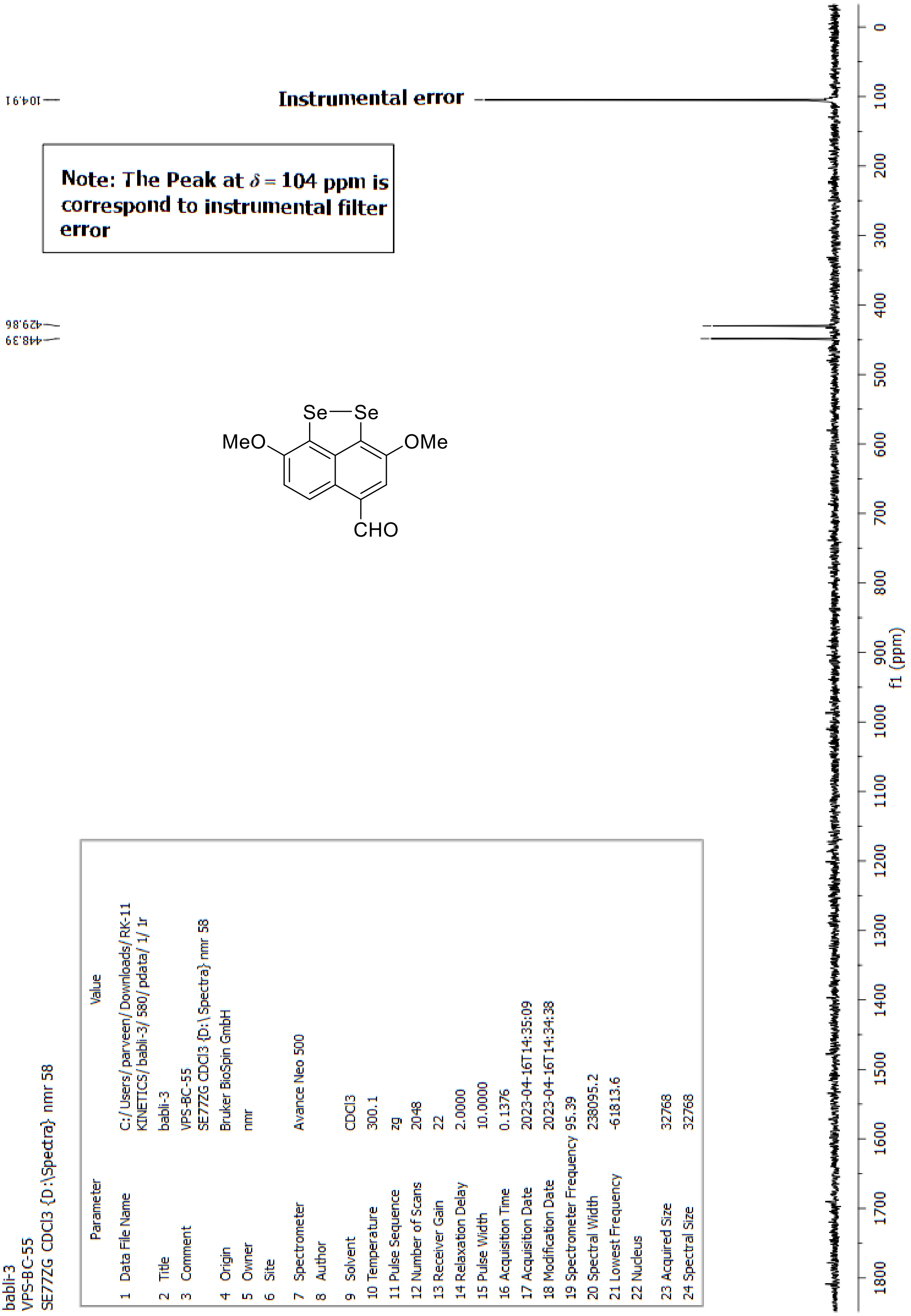

Figure S4. HRMS data of compound **7**

Department of Chemistry I.I.T. (B)

Agilent Trusted Answers

Sample Information

**Name**	EXT-PUNJAB UNI-VPS-BC-55	**Data File Path**	D:\MassHunter\Data\DEC-21\EXT-PUNJAB UNI-VPS-BC-55.d
**Sample ID**		**Acq. Time (Local)**	23-12-2021 1.04.39 PM (UTC+05:30)
**Instrument**	LCMS QTOF	**Method Path (Acq)**	D:\MassHunter\Methods\6545XT checkout\Methods\TRAINING\MS SCAN_AB_POS_4000-1000-150.m
**MS Type**	QTOF	**Version (Acq SW)**	6200 series TOF/6500 series Q-TOF B.09.00 (B9044.0)
**Inj. Vol. (ul)**	1	**IRM Status**	Success
**Position**	P1-C3	**Method Path (DA)**	D:\MassHunter\Report Templates\REPORT METHOD\HRMS_IITB.m
**Plate Pos.**		**Target Source Path**	
**Operator**	RAJESH	**Result Summary**	1 qualified (1 targets)

Sample Spectra

+ Scan (rt: 0.228-0.494 min) **Peak 1 from + TIC Scan**

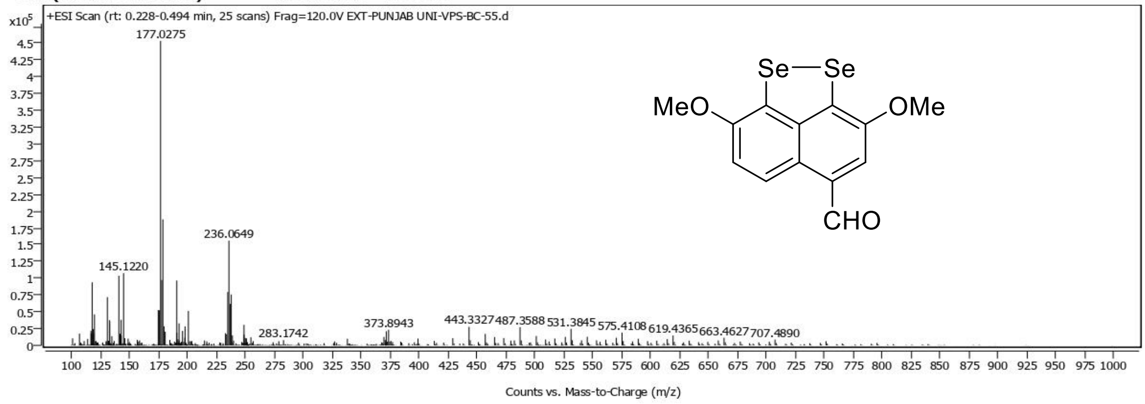

Compound Details

Cpd. 1: C13 H10 O3 Se2

Formula	m/z	Observed M/Z	Difference_Da	Difference_PPM	Score
C13 H10 O3 Se2	373.8943	373.894257158622	-1.41310995473987	-3.90461155707132	65.33

Compound Spectra (Zoomed)

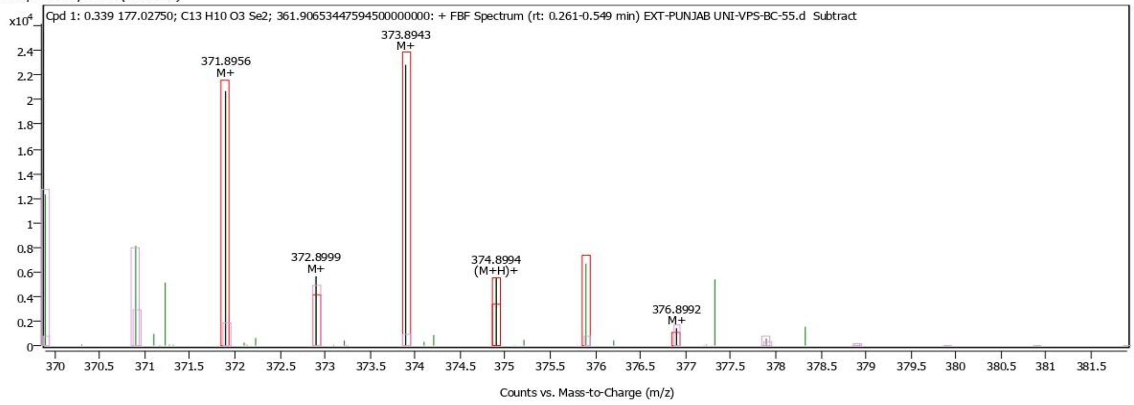

MassHunter Qual 10.0
(End of Report)

MassHunter Qualitative Analysis | Page 1 of 1 | Generated at 04.05 PM on 03-01-2022

Figure S5. ^{1}H NMR spectrum of compound **8** in DMSO-d_6

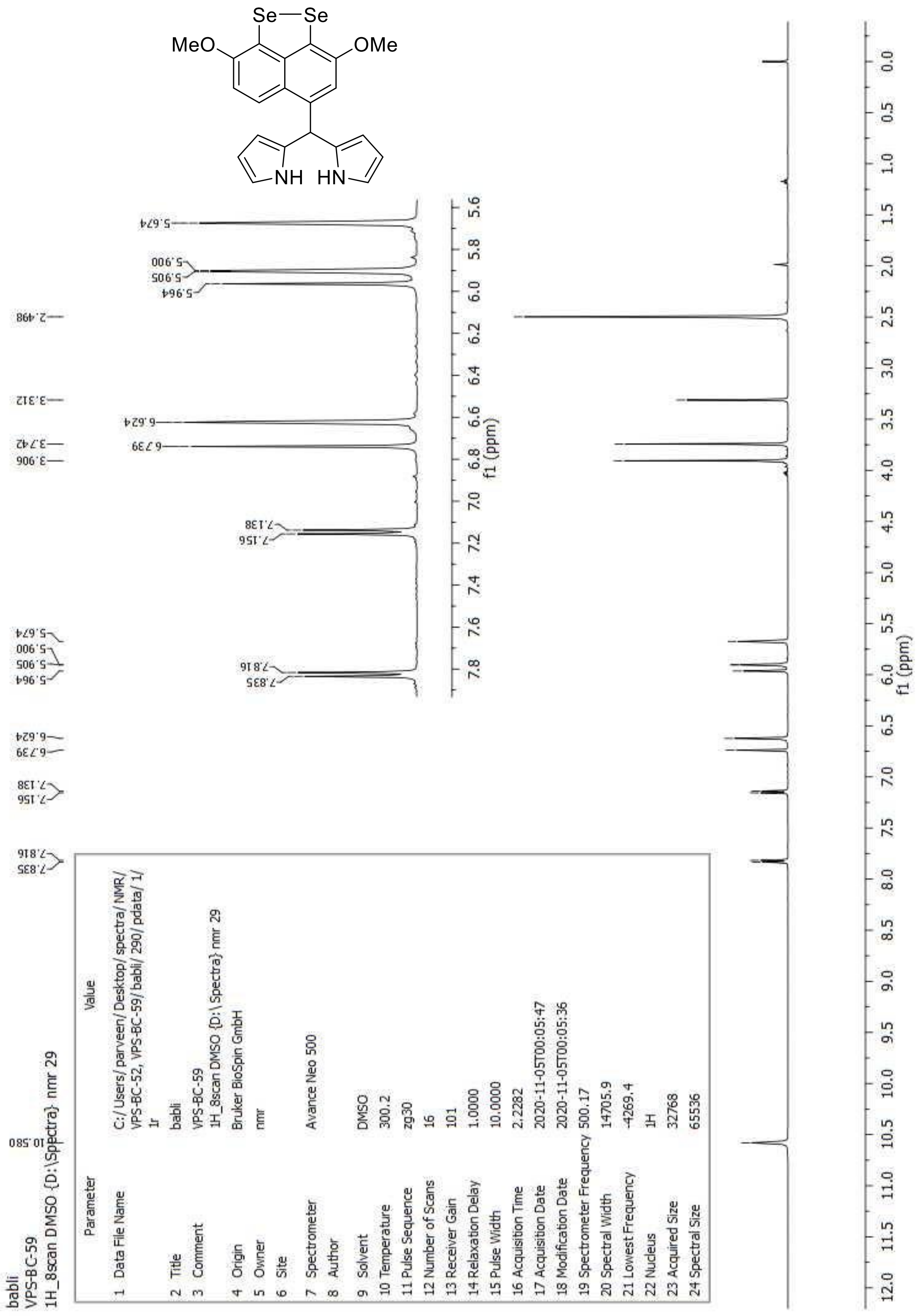

Figure S6. $^{13}C\{^1H\}$ NMR spectrum of compound **8** in DMSO-d_6

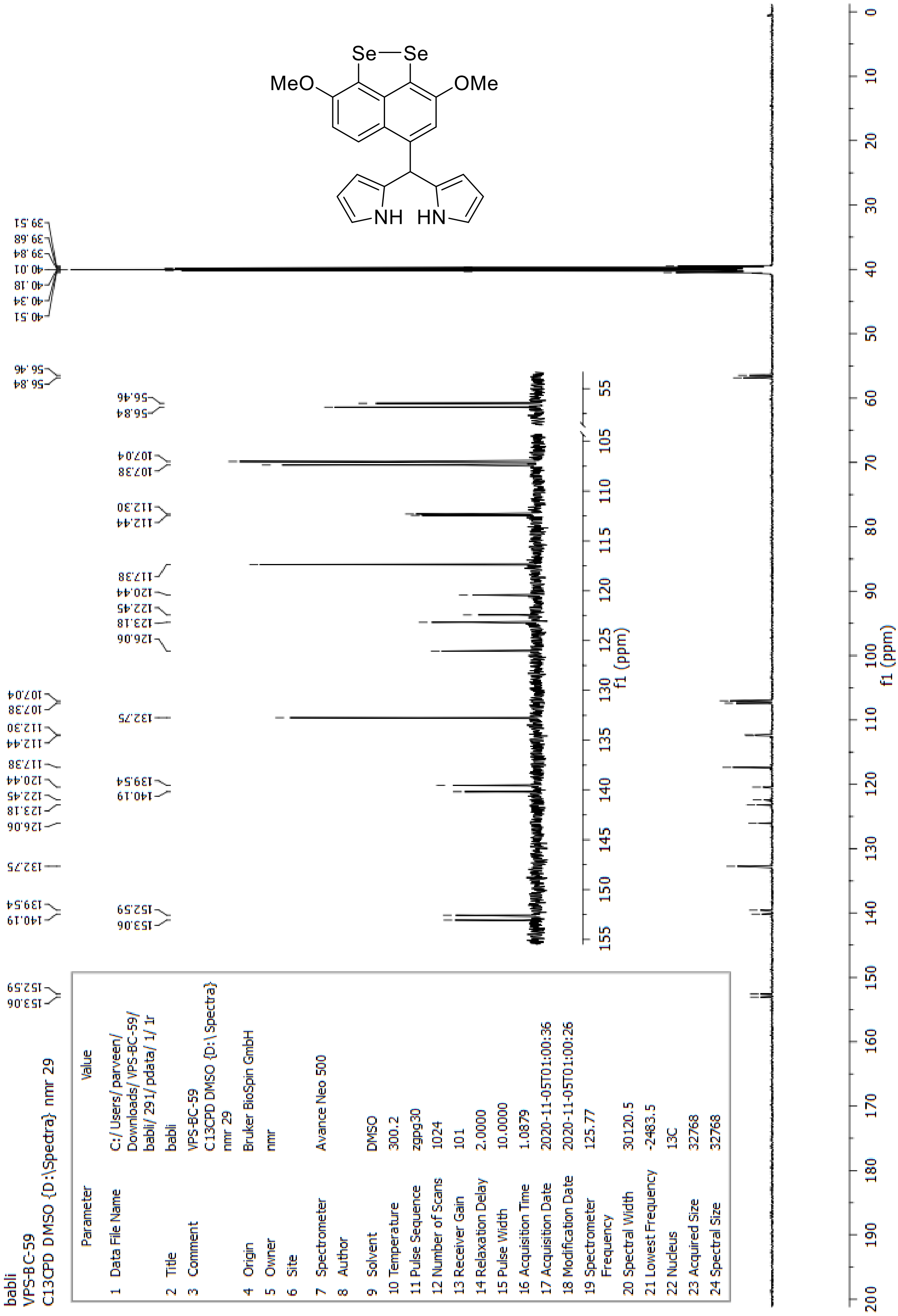

Figure S7. $^{77}Se\{^1H\}$ NMR spectrum of compound **8** in DMSO-d_6

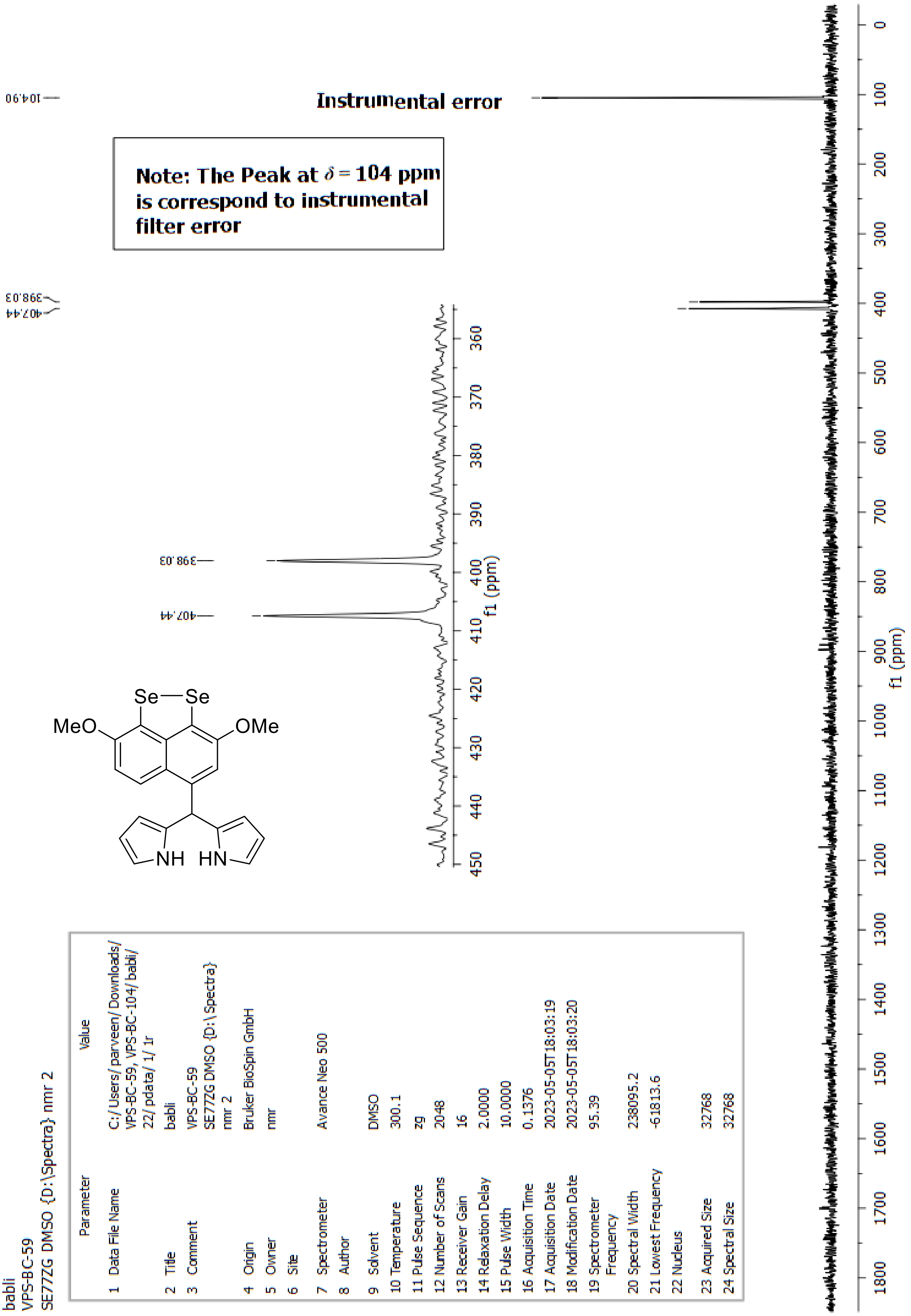

Figure S8. HRMS data of compound **8**

Department of Chemistry I.I.T. (B)

Agilent | Trusted Answers

Sample Information

**Name**	EXT-PUNJAB UNI-VPS-BC-59	**Data File Path**	D:\MassHunter\Data\DEC-21\EXT-PUNJAB UNI-VPS-BC-59.d
**Sample ID**		**Acq. Time (Local)**	23-12-2021 1.12.41 PM (UTC+05:30)
**Instrument**	LCMS QTOF	**Method Path (Acq)**	D:\MassHunter\Methods\6545XT checkout\Methods\TRAINING\MS SCAN_AB_POS_4000-1000-150.m
**MS Type**	QTOF	**Version (Acq SW)**	6200 series TOF/6500 series Q-TOF B.09.00 (B9044.0)
**Inj. Vol. (ul)**	1	**IRM Status**	Success
**Position**	P1-C6	**Method Path (DA)**	D:\MassHunter\Report Templates\REPORT METHOD\HRMS_IITB.m
**Plate Pos.**		**Target Source Path**	
**Operator**	RAJESH	**Result Summary**	1 qualified (1 targets)

Sample Spectra

+ Scan (rt: 0.247-0.612 min) **Peak 1 from + TIC Scan**

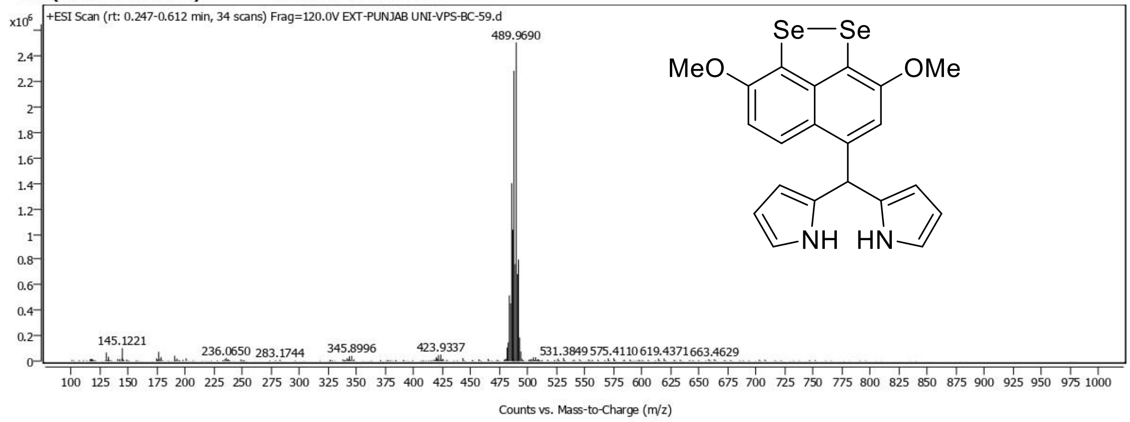

Compound Details

Cpd. 1: C21 H18 N2 O2 Se2

Formula	m/z	Observed M/Z	Difference_Da	Difference_PPM	Score
C21 H18 N2 O2 Se2	489.9689	489.968943034632	-1.0168941144002	-2.12747463255974	83.85

Compound Spectra (Zoomed)

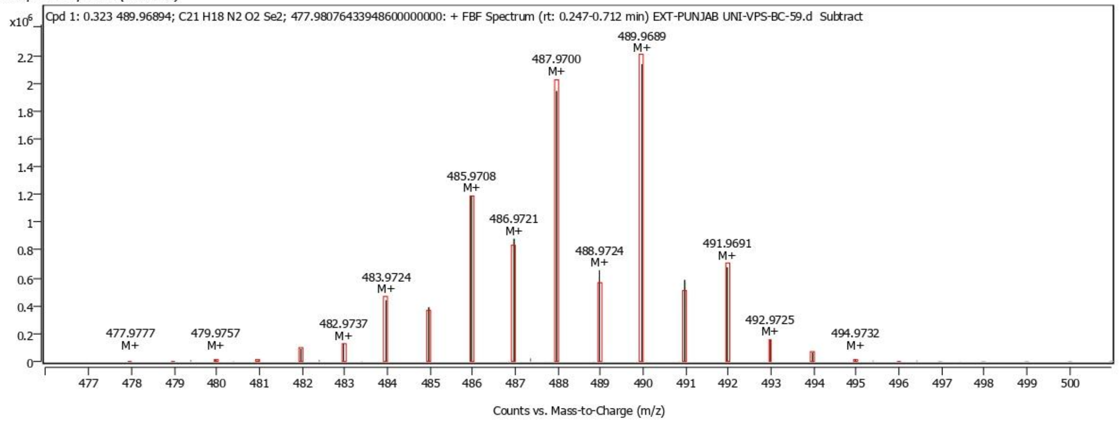

MassHunter Qual 10.0
(End of Report)

MassHunter Qualitative Analysis | Page 1 of 1 | Generated at 04.07 PM on 03-01-2022

Figure S9. ^{1}H NMR spectrum of compound **9** in DMSO-d_6

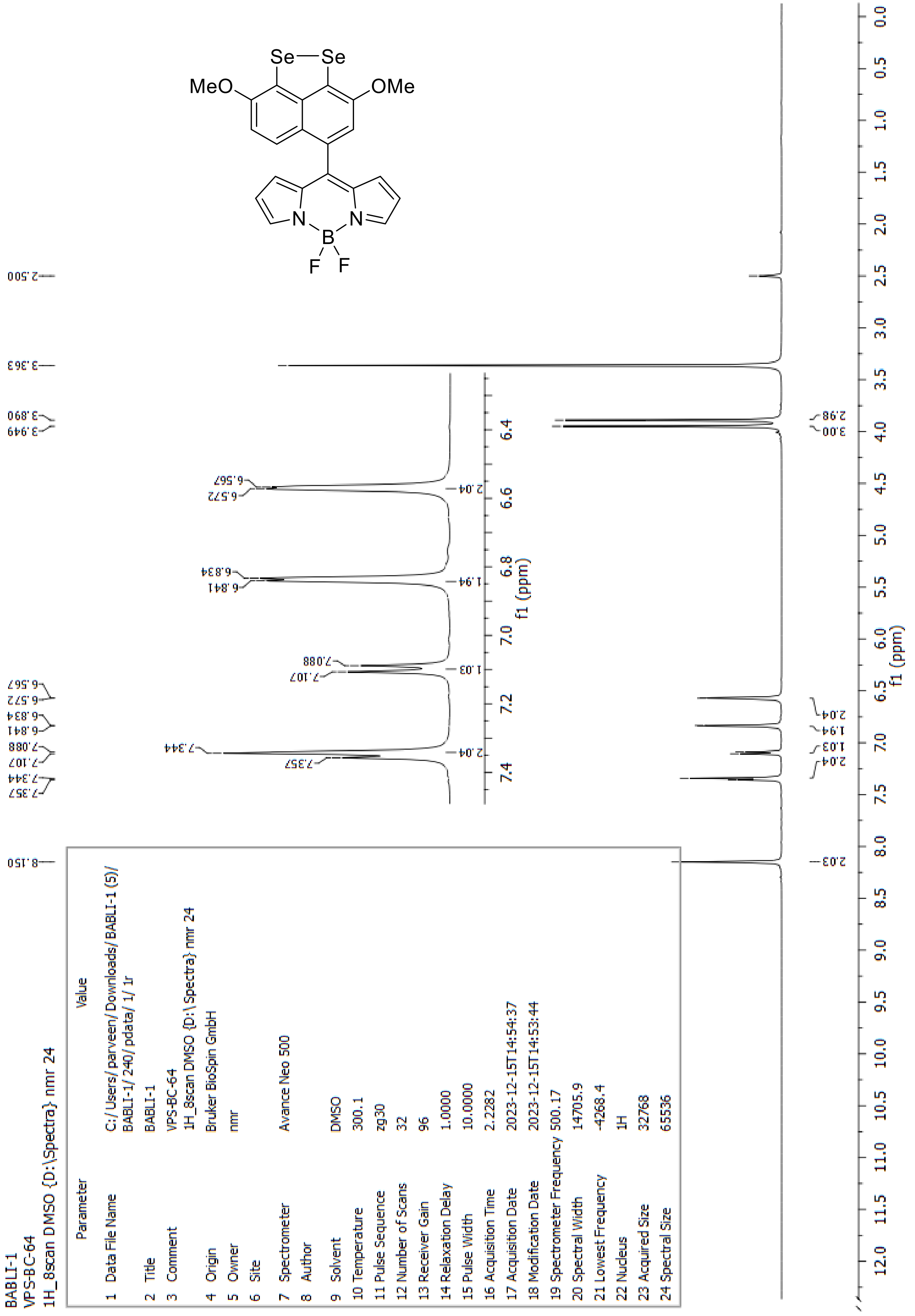

Figure S10. $^{13}C\{^{1}H\}$ NMR spectrum of compound **9** in DMSO-d_6

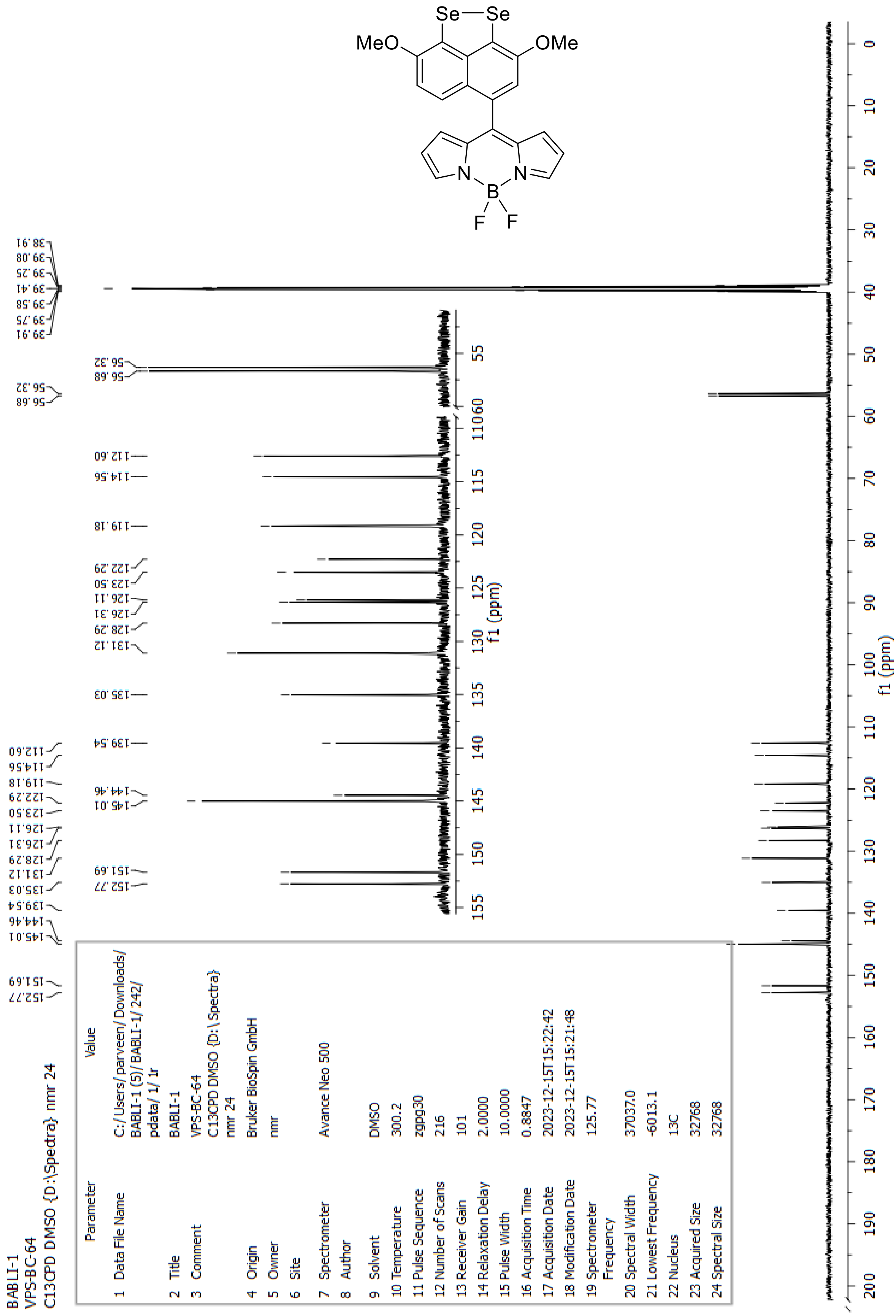

Figure S11. $^{77}Se\{^{1}H\}$ NMR spectrum of probe **9** in DMSO-d_6

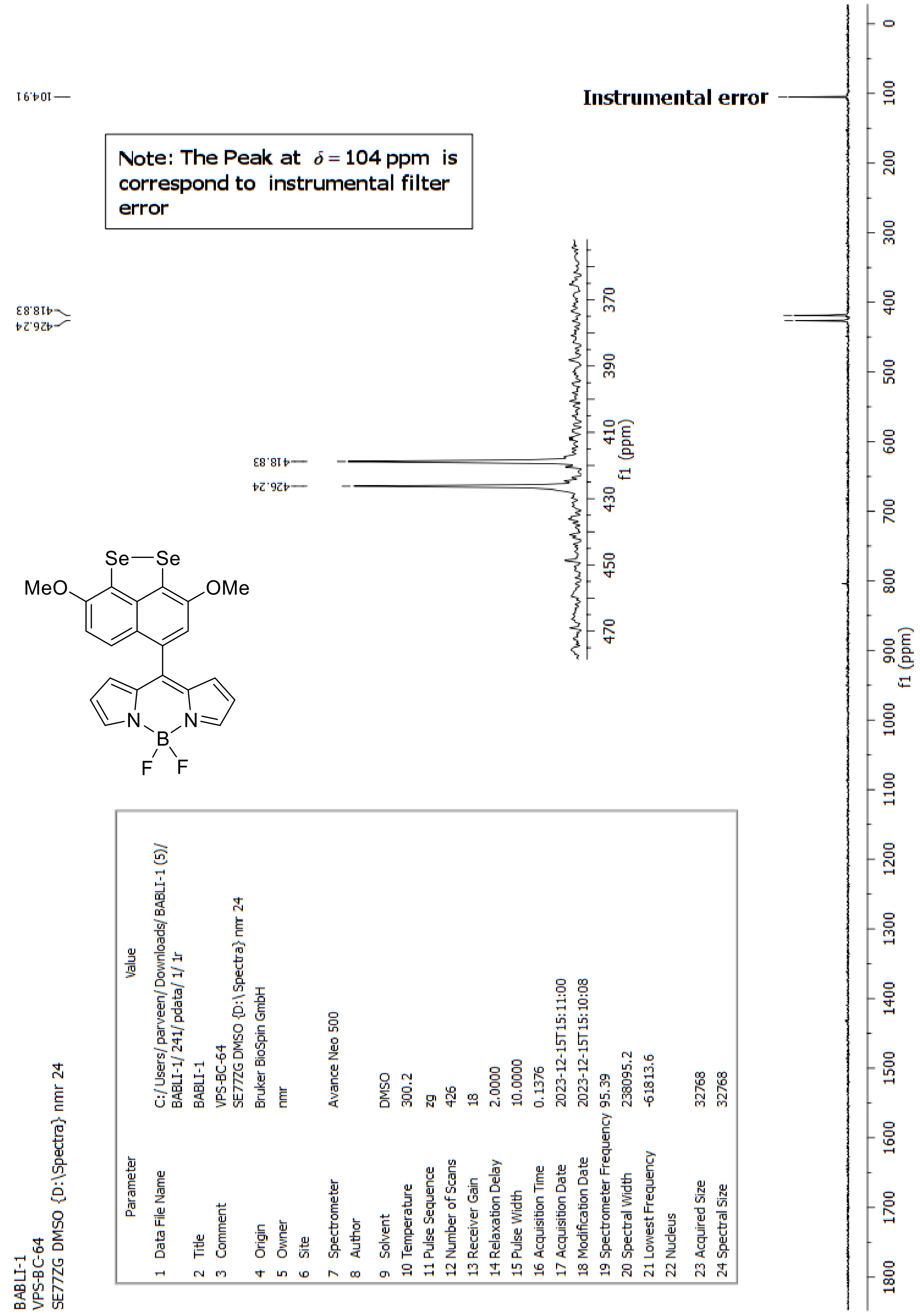

Figure S12. HRMS data of compound **9**

Department of Chemistry I.I.T. (B)

Agilent | Trusted Answers

Sample Information

**Name**	EXT-PUNJAB UNI-VPS-BC-64	**Data File Path**	D:\MassHunter\Data\DEC-21\EXT-PUNJAB UNI-VPS-BC-64.d
**Sample ID**		**Acq. Time (Local)**	23-12-2021 1.10.00 PM (UTC+05:30)
**Instrument**	LCMS QTOF	**Method Path (Acq)**	D:\MassHunter\Methods\6545XT checkout\Methods\TRAINING\MS SCAN_AB_POS_4000-1000-150.m
**MS Type**	QTOF	**Version (Acq SW)**	6200 series TOF/6500 series Q-TOF B.09.00 (B9044.0)
**Inj. Vol. (ul)**	1	**IRM Status**	Success
**Position**	P1-C5	**Method Path (DA)**	D:\MassHunter\Report Templates\REPORT METHOD\HRMS_IITB.m
**Plate Pos.**		**Target Source Path**	
**Operator**	RAJESH	**Result Summary**	1 qualified (1 targets)

Sample Spectra

+ Scan (rt: 0.231-0.542 min) **Peak 1 from + TIC Scan**

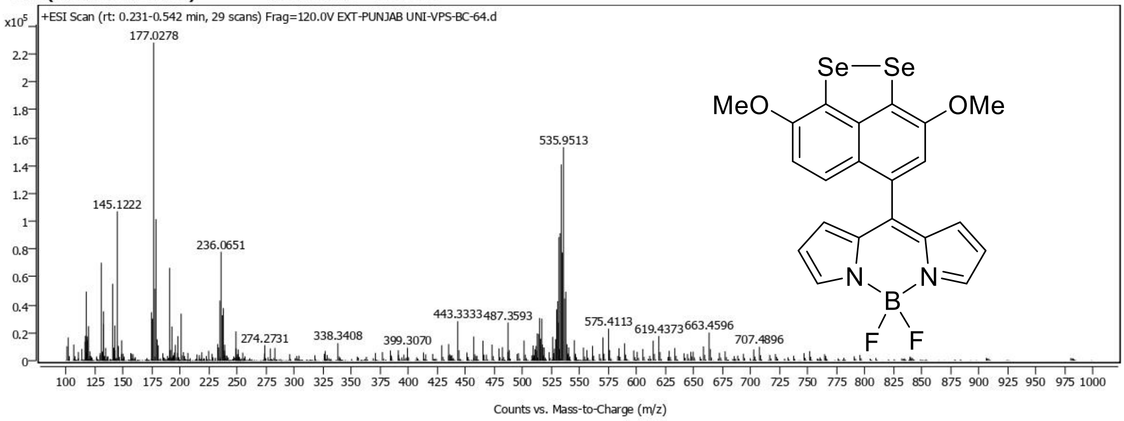

Compound Details

Cpd. 1: C21 H15 B F2 N2 O2 Se2

Formula	m/z	Observed M/Z	Difference_Da	Difference_PPM	Score
C21 H15 B F2 N2 O2 Se2	535.9513	535.951292780147	-1.79850897995948	-3.43904179462733	67.58

Compound Spectra (Zoomed)

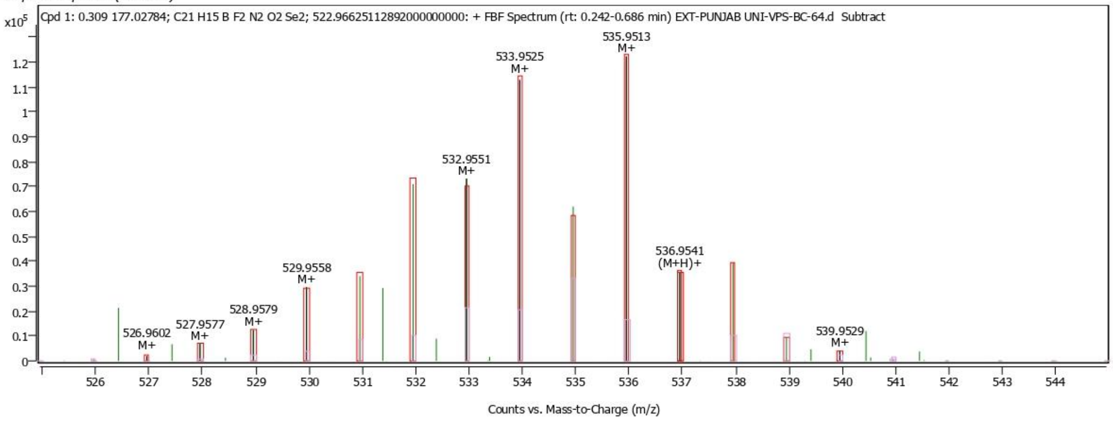

MassHunter Qual 10.0
(End of Report)

Figure S13. FTIR analysis of probe **9**

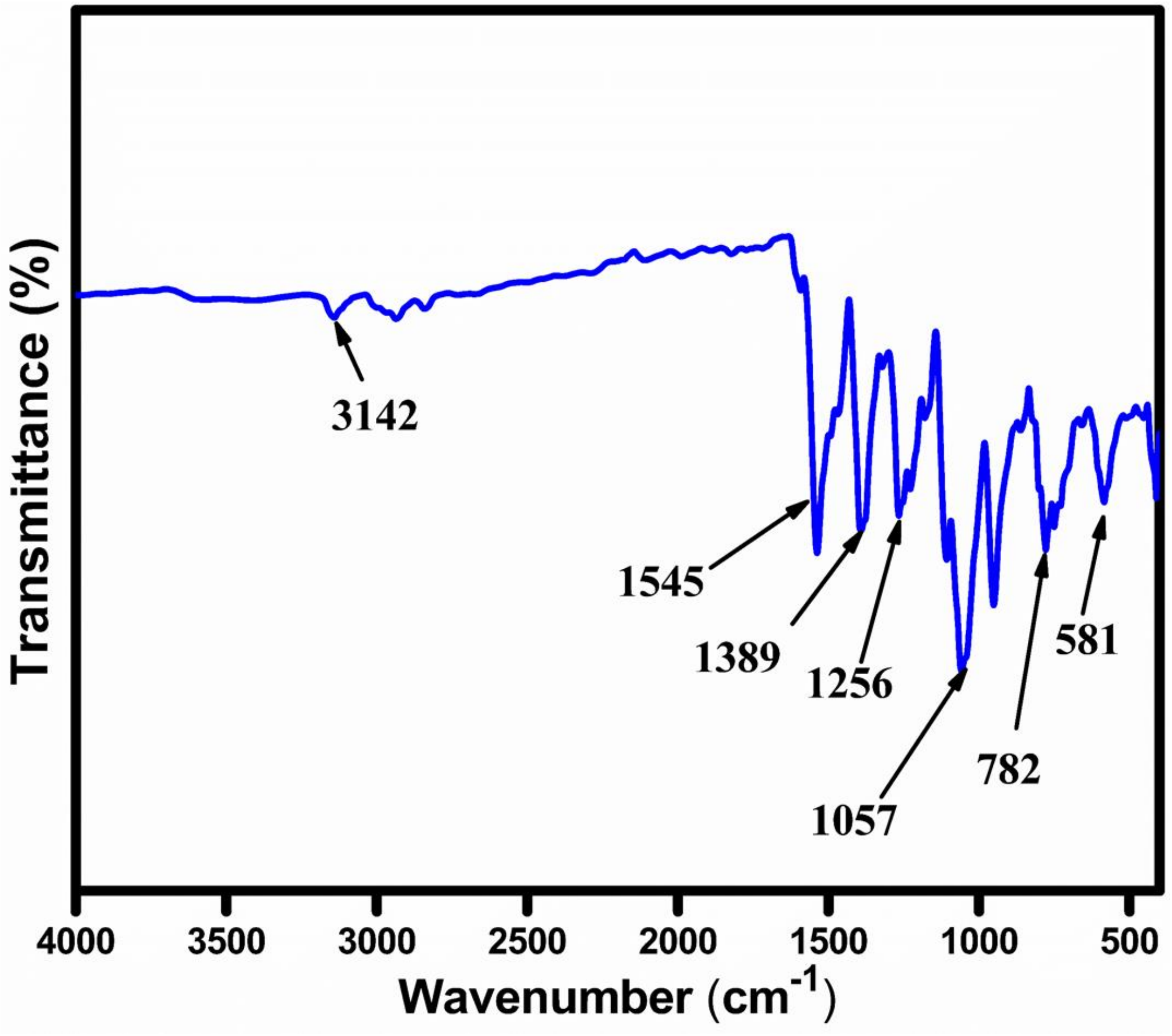

Table S1. Crystal Data and Structure Refinement for Compound **6**.

Identification code	**VPS-BC-6**
Empirical formula	$C_{24}H_{20}O_4Se_4$
Formula weight	688.24
Temperature/K	293(2)
Crystal system	monoclinic
Space group	$P2_1/n$
a/Å	11.9484(3)
b/Å	12.3700(4)
c/Å	16.1224(4)
α/°	90
β/°	102.636(3)
γ/°	90
Volume/Å^3	2325.20(11)
Z	4
ρ_{calc}g/cm^3	1.966
μ/mm^{-1}	6.341
F(000)	1328.0
Radiation	Mo Kα (λ = 0.71073)
2Θ range for data collection/°	6.478 to 54.802
Index ranges	$-14 \le h \le 14$, $-15 \le k \le 15$, $-19 \le l \le 20$
Reflections collected	30471
Independent reflections	5009 [R_{int} = 0.0909, R_{sigma} = 0.0925]
Data/restraints/parameters	5009/0/293
Goodness-of-fit on F^2	1.067
Final R indexes [I>=2σ (I)]	R_1 = 0.0742, wR_2 = 0.1196
Final R indexes [all data]	R_1 = 0.1403, wR_2 = 0.1367
Largest diff. peak/hole / e Å^{-3}	0.64/-0.73

Table S2. Fractional Atomic Coordinates (×10^4) and Equivalent Isotropic Displacement Parameters (Å^2×10^3) for Compound **6**. U_{eq} is defined as 1/3 of the trace of the orthogonalised U_{IJ} tensor.

**Atom**	***x***	***Y***	***z***	**U(eq)**
Se1	-567.6(6)	6052.6(7)	1843.0(4)	52.2(3)
Se2	587.9(6)	7039.6(7)	2962.1(5)	50.9(2)
Se3	6193.7(7)	5694.1(6)	6337.8(5)	57.4(3)
Se4	8026.8(7)	5832.7(6)	7249.6(5)	56.8(3)
O2	938(4)	7467(5)	4807(3)	63.8(15)
O1	-2566(4)	4600(4)	1433(3)	58.1(14)
O4	9966(4)	7341(4)	7816(3)	61.2(14)
O3	4555(4)	6850(4)	4995(3)	64.2(15)
C3	-226(5)	6446(6)	3756(4)	40.7(17)
C1	-1444(5)	5459(5)	2586(4)	38.3(16)
C26	9149(6)	7874(6)	7233(4)	46.7(18)
C2	-1133(5)	5742(5)	3455(4)	36.9(16)
C24	8406(6)	9392(5)	6356(4)	42.6(17)
C18	7300(6)	7722(6)	6271(4)	38.8(17)
C4	54(6)	6765(6)	4604(4)	45.4(18)
C17	8171(6)	7280(5)	6890(4)	40.2(17)
C23	7407(6)	8798(6)	5973(4)	48.6(19)
C22	6532(6)	9209(6)	5324(4)	47.2(19)
C20	5464(6)	7531(6)	5283(4)	45.3(18)
C10	-2348(6)	4795(6)	2290(4)	44.2(18)
C19	6303(6)	7111(6)	5912(4)	41.6(17)
C7	-1775(6)	5323(6)	4026(4)	45.7(18)
C25	9232(7)	8940(7)	6951(5)	55(2)
C8	-2686(6)	4619(6)	3696(5)	53(2)
C9	-2971(6)	4356(6)	2856(5)	53(2)
C6	-1476(7)	5649(6)	4879(5)	55(2)
C21	5586(7)	8578(7)	4986(4)	55(2)
C5	-587(6)	6342(6)	5159(4)	54(2)
C32	10986(7)	7898(8)	8199(5)	76(3)
C30	3542(6)	7274(8)	4458(5)	71(3)
C14	1182(7)	7928(8)	5638(5)	84(3)
C16	-3542(7)	3961(8)	1068(5)	82(3)

Table S3. Anisotropic Displacement Parameters (Å^2×10^3) for Compound **6**. The Anisotropic displacement factor exponent takes the form: $2\pi^2[h^2a^{*2}U_{11}+2hka^*b^*U_{12}+\ldots]$.

Atom	U_{11}	U_{22}	U_{33}	U_{23}	U_{13}	U_{12}
Se1	45.8(4)	74.0(6)	38.0(4)	4.7(4)	12.0(3)	-9.6(4)
Se2	43.3(4)	59.8(6)	51.7(5)	-1.0(4)	14.8(4)	-8.6(4)
Se3	52.6(5)	49.2(5)	69.0(6)	8.7(4)	10.1(4)	-7.4(4)
Se4	59.4(5)	49.1(5)	59.0(5)	11.9(4)	6.3(4)	-2.0(4)
O2	51(3)	86(4)	52(3)	-26(3)	5(3)	-5(3)
O1	54(3)	65(4)	54(3)	-6(3)	9(3)	-18(3)
O4	50(3)	65(4)	65(3)	10(3)	4(3)	-4(3)
O3	46(3)	68(4)	72(4)	6(3)	-2(3)	0(3)
C3	32(4)	50(5)	42(4)	5(4)	13(3)	11(3)
C1	33(4)	38(4)	45(4)	1(3)	11(3)	-5(3)
C26	48(5)	50(5)	47(4)	-1(4)	20(4)	0(4)
C2	33(4)	37(4)	41(4)	6(3)	8(3)	10(3)
C24	52(5)	27(4)	54(5)	-3(3)	23(4)	-3(4)
C18	43(4)	39(5)	40(4)	-7(3)	20(3)	2(3)
C4	37(4)	52(5)	44(4)	-6(4)	3(4)	14(4)
C17	45(4)	40(4)	40(4)	-1(3)	18(3)	0(3)
C23	61(5)	42(5)	51(5)	-1(4)	31(4)	8(4)
C22	51(5)	39(5)	56(5)	11(4)	19(4)	12(4)
C20	43(4)	53(5)	42(4)	0(4)	15(4)	6(4)
C10	45(4)	45(5)	42(4)	9(4)	10(4)	6(4)
C19	51(4)	42(5)	32(4)	1(3)	11(3)	6(4)
C7	42(4)	49(5)	48(5)	5(4)	15(4)	7(4)
C25	56(5)	48(5)	66(5)	-5(4)	24(4)	-12(4)
C8	50(5)	64(6)	50(5)	15(4)	24(4)	6(4)
C9	50(5)	40(5)	73(6)	8(4)	20(4)	-11(4)
C6	56(5)	69(6)	45(5)	13(4)	20(4)	15(4)
C21	66(5)	57(6)	44(4)	6(4)	18(4)	19(4)
C5	54(5)	72(6)	39(4)	-5(4)	15(4)	20(4)
C32	48(5)	93(7)	83(6)	4(5)	5(4)	-20(5)
C30	41(5)	103(8)	66(5)	10(5)	5(4)	4(5)
C14	71(6)	106(8)	67(6)	-35(6)	1(5)	10(5)
C16	66(6)	100(8)	79(6)	-35(6)	11(5)	-27(5)

Table S4. Bond Lengths for Compound **6**.

Atom	Atom	Length/Å	Atom	Atom	Length/Å
Se1	Se2	2.3586(11)	C26	C17	1.389(9)
Se1	C1	1.902(6)	C26	C25	1.404(10)
Se2	C3	1.915(6)	C2	C7	1.418(9)
Se3	Se4	2.3619(11)	C24	C23	1.423(10)
Se3	C19	1.897(7)	C24	C25	1.339(10)
Se4	C17	1.901(7)	C18	C17	1.387(9)
O2	C4	1.352(8)	C18	C23	1.430(9)
O2	C14	1.427(8)	C18	C19	1.422(9)
O1	C10	1.371(7)	C4	C5	1.401(10)
O1	C16	1.425(8)	C23	C22	1.403(9)
O4	C26	1.367(8)	C22	C21	1.383(10)
O4	C32	1.419(9)	C20	C19	1.363(9)
O3	C20	1.373(8)	C20	C21	1.399(10)
O3	C30	1.425(8)	C10	C9	1.407(9)
C3	C2	1.392(9)	C7	C8	1.405(10)
C3	C4	1.392(9)	C7	C6	1.402(10)
C1	C2	1.413(8)	C8	C9	1.362(9)
C1	C10	1.358(9)	C6	C5	1.363(10)

Table S5. Bond Angles for Compound **6**.

Atom	Atom	Atom	Angle/°	Atom	Atom	Atom	Angle/°
C1	Se1	Se2	91.6(2)	C3	C4	C5	118.4(7)
C3	Se2	Se1	90.8(2)	C26	C17	Se4	120.1(5)
C19	Se3	Se4	91.7(2)	C18	C17	Se4	119.1(5)
C17	Se4	Se3	90.7(2)	C18	C17	C26	120.7(6)
C4	O2	C14	118.2(6)	C24	C23	C18	117.6(7)
C10	O1	C16	118.1(6)	C22	C23	C24	123.6(7)
C26	O4	C32	118.7(6)	C22	C23	C18	118.8(7)
C20	O3	C30	118.9(6)	C21	C22	C23	120.1(7)
C2	C3	Se2	118.6(5)	O3	C20	C21	125.9(7)
C4	C3	Se2	120.0(5)	C19	C20	O3	114.6(7)
C4	C3	C2	121.3(6)	C19	C20	C21	119.5(7)
C2	C1	Se1	117.9(5)	O1	C10	C9	125.1(7)
C10	C1	Se1	121.2(5)	C1	C10	O1	114.9(6)
C10	C1	C2	121.0(6)	C1	C10	C9	119.9(6)
O4	C26	C17	115.4(7)	C18	C19	Se3	117.5(5)
O4	C26	C25	125.8(7)	C20	C19	Se3	121.6(6)
C17	C26	C25	118.8(7)	C20	C19	C18	120.9(7)
C3	C2	C1	121.0(6)	C8	C7	C2	117.7(6)
C3	C2	C7	119.6(6)	C6	C7	C2	118.3(7)
C1	C2	C7	119.4(6)	C6	C7	C8	124.0(7)
C25	C24	C23	120.8(7)	C24	C25	C26	122.0(7)
C17	C18	C23	120.0(6)	C9	C8	C7	121.9(7)
C17	C18	C19	120.9(6)	C8	C9	C10	120.0(7)
C19	C18	C23	119.1(6)	C5	C6	C7	121.3(7)
O2	C4	C3	115.3(6)	C22	C21	C20	121.6(7)
O2	C4	C5	126.4(7)	C6	C5	C4	121.2(7)

Table S6. Torsion Angles for Compound **6**.

A	B	C	D	Angle/°	A	B	C	D	Angle/°
Se1	C1	C2	C3	0.9(8)	C17	C18	C23	C24	-2.3(9)
Se1	C1	C2	C7	179.4(5)	C17	C18	C23	C22	177.6(6)
Se1	C1	C10	O1	-0.1(8)	C17	C18	C19	Se3	2.0(8)
Se1	C1	C10	C9	179.1(5)	C17	C18	C19	C20	-177.7(6)
Se2	C3	C2	C1	0.7(8)	C23	C24	C25	C26	-0.8(10)
Se2	C3	C2	C7	-177.8(5)	C23	C18	C17	Se4	-178.1(4)
Se2	C3	C4	O2	-2.2(8)	C23	C18	C17	C26	0.6(9)
Se2	C3	C4	C5	177.8(5)	C23	C18	C19	Se3	-179.3(4)
Se4	Se3	C19	C18	-2.8(5)	C23	C18	C19	C20	0.9(9)
Se4	Se3	C19	C20	176.9(5)	C23	C22	C21	C20	0.8(10)
O2	C4	C5	C6	179.0(7)	C10	C1	C2	C3	-178.2(6)
O1	C10	C9	C8	-179.1(7)	C10	C1	C2	C7	0.3(10)
O4	C26	C17	Se4	0.8(8)	C19	C18	C17	Se4	0.5(8)
O4	C26	C17	C18	-177.8(5)	C19	C18	C17	C26	179.2(6)
O4	C26	C25	C24	177.8(6)	C19	C18	C23	C24	179.0(6)
O3	C20	C19	Se3	0.0(8)	C19	C18	C23	C22	-1.1(9)
O3	C20	C19	C18	179.8(5)	C19	C20	C21	C22	-1.0(10)
O3	C20	C21	C22	179.4(6)	C7	C8	C9	C10	-0.2(11)
C3	C2	C7	C8	179.7(6)	C7	C6	C5	C4	0.6(11)
C3	C2	C7	C6	0.8(10)	C25	C26	C17	Se4	179.7(5)
C3	C4	C5	C6	-1.0(11)	C25	C26	C17	C18	1.1(9)
C1	C2	C7	C8	1.2(10)	C25	C24	C23	C18	2.5(9)
C1	C2	C7	C6	-177.7(6)	C25	C24	C23	C22	-177.5(6)
C1	C10	C9	C8	1.7(11)	C8	C7	C6	C5	-179.4(7)
C2	C3	C4	O2	-178.7(6)	C6	C7	C8	C9	177.5(7)
C2	C3	C4	C5	1.3(10)	C21	C20	C19	Se3	-179.7(5)
C2	C1	C10	O1	179.0(6)	C21	C20	C19	C18	0.1(9)
C2	C1	C10	C9	-1.8(10)	C32	O4	C26	C17	-179.5(6)
C2	C7	C8	C9	-1.3(11)	C32	O4	C26	C25	1.7(10)
C2	C7	C6	C5	-0.6(10)	C30	O3	C20	C19	168.3(6)
C24	C23	C22	C21	-179.9(6)	C30	O3	C20	C21	-12.0(10)
C18	C23	C22	C21	0.2(9)	C14	O2	C4	C3	172.5(6)
C4	C3	C2	C1	177.3(6)	C14	O2	C4	C5	-7.5(11)
C4	C3	C2	C7	-1.2(10)	C16	O1	C10	C1	-176.4(7)
C17	C26	C25	C24	-1.0(10)	C16	O1	C10	C9	4.4(10)

Table S7. Hydrogen Atom Coordinates (Å×10^4) and Isotropic Displacement Parameters (Å^2×10^3) for Compound **6**.

Atom	x	Y	z	U(eq)
H24	8486.39	10103.32	6191.29	51
H22	6588	9905.47	5120.65	57
H25	9880.75	9344.12	7185.69	66
H8	-3106.95	4323.18	4062.38	63
H9	-3577.75	3886.03	2656.42	64
H6	-1891.37	5386.17	5260.86	66
H21	5016.21	8857.09	4551.38	66
H5	-403.13	6538.97	5729.83	65
H32A	11376.48	8124.37	7768.16	114
H32B	11474.84	7426.3	8592.54	114
H32C	10796.14	8520.17	8496.95	114
H30A	3700.83	7473.45	3920.17	106
H30B	3292.89	7899.52	4720.97	106
H30C	2949.52	6735.22	4372.36	106
H14A	1831.68	8401.75	5698.32	125
H14B	1349.89	7362.58	6054.24	125
H14C	528.66	8331.54	5720.89	125
H16A	-3564.35	3848.57	475.28	123
H16B	-4226.71	4329.65	1129.35	123
H16C	-3493.59	3276.04	1352.72	123

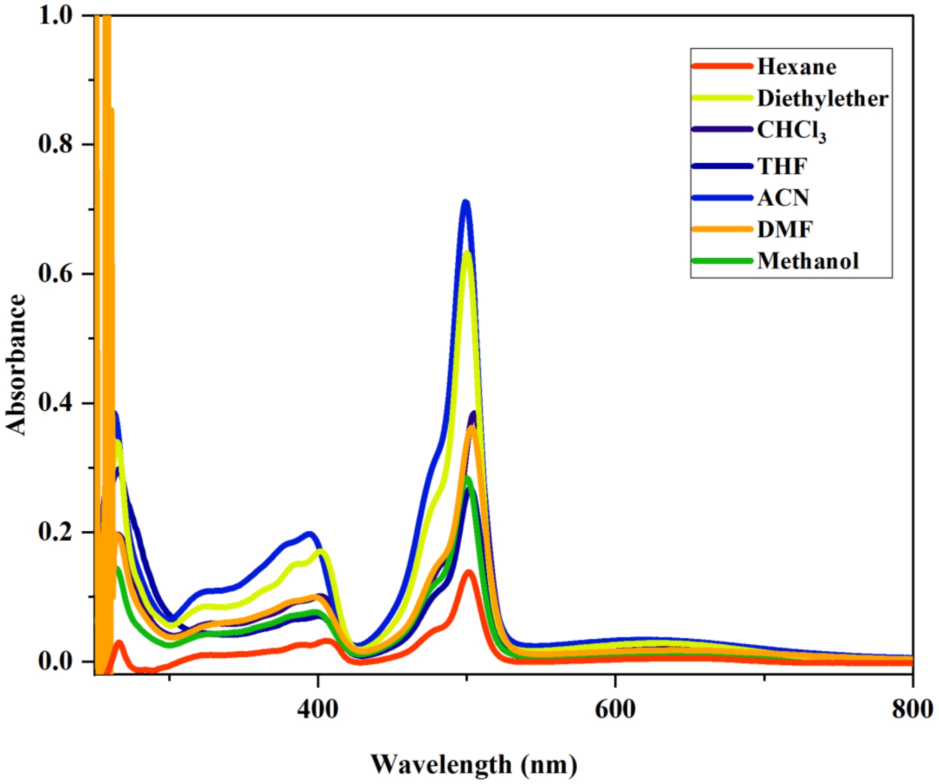

Figure S14. The absorption solvatochromic studies of the probe **9** (10 μM) with various solvents

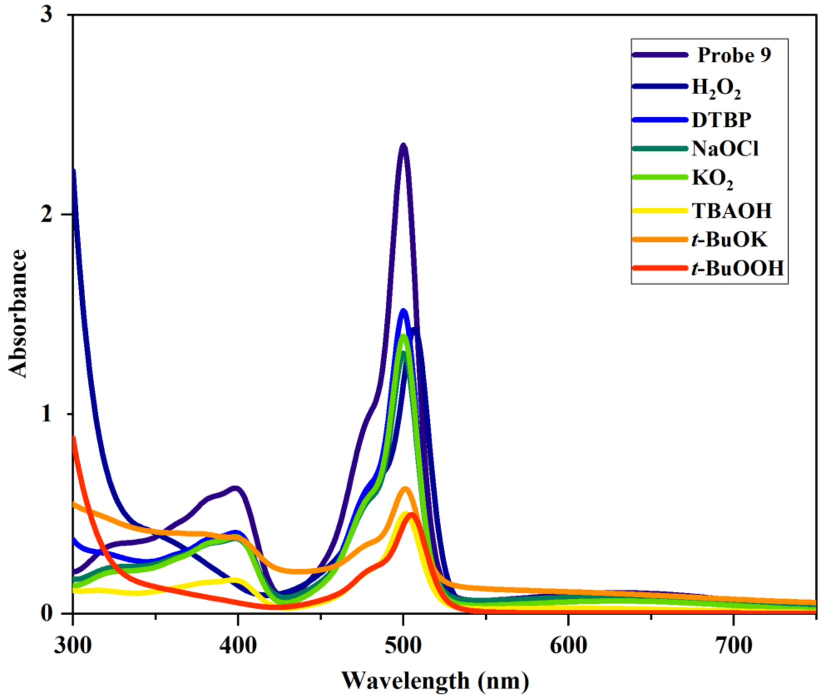

Figure S15. UV-Vis spectral changes of the probe **9** (10 µM, methanol/water: v/v = 70:30) with various ROSs (1.20 M)

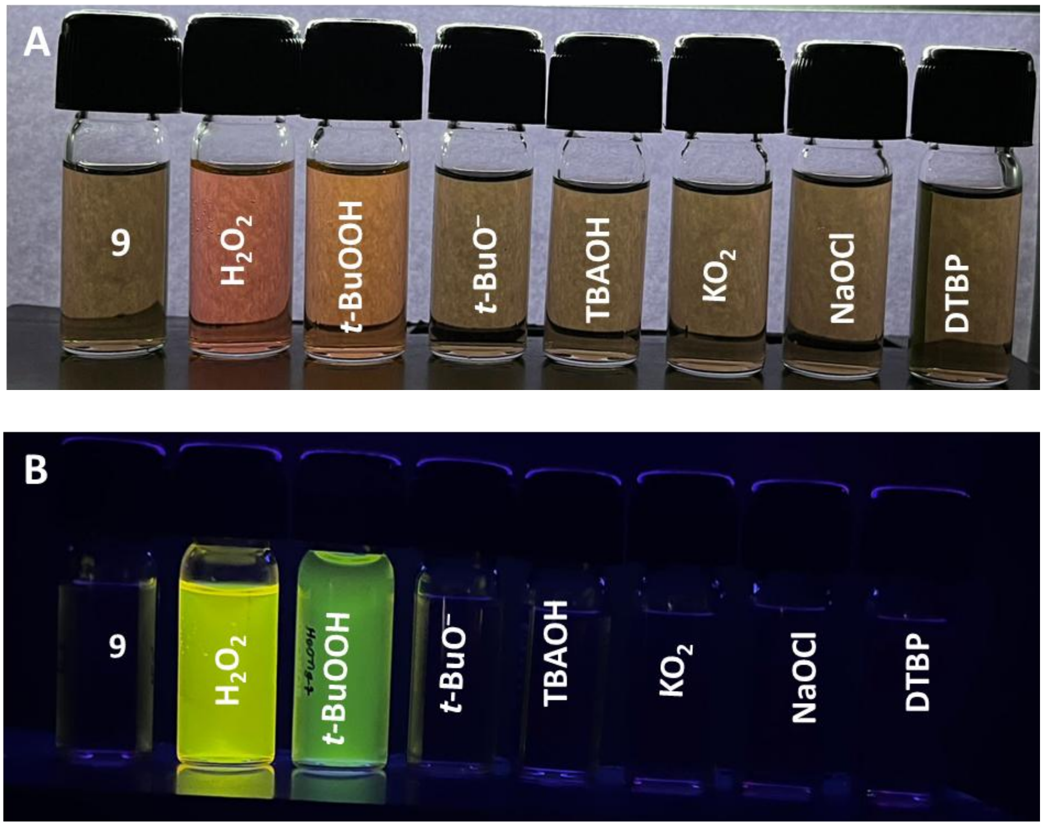

Figure S16. Digital photos of probes **9**, probes **9** + H_2O_2, probes **9** + *t*-BuOOH and probes **9** + other ROS (1.0 M) taken under (A) natural light and (B) UV light

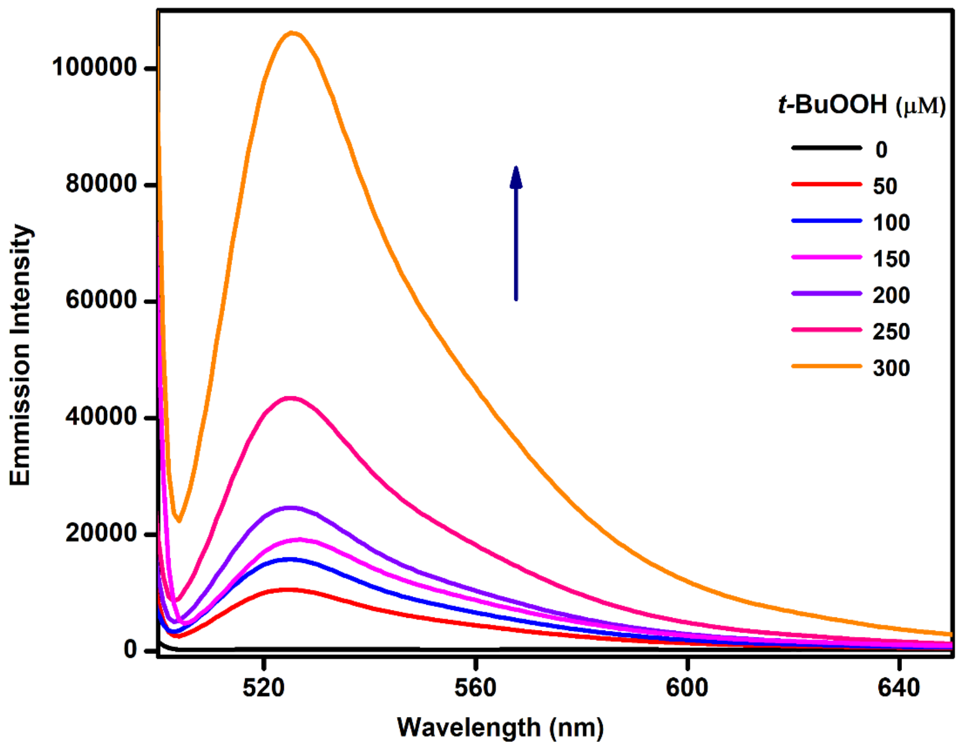

Figure S17. The emission spectra of **9** (10 µM, methanol/water: v/v = 70:30) with increasing concentrations of t-BuOOH (0–300 µM); (λ_{ex} = 485 nm and λ_{em} = 525 nm).

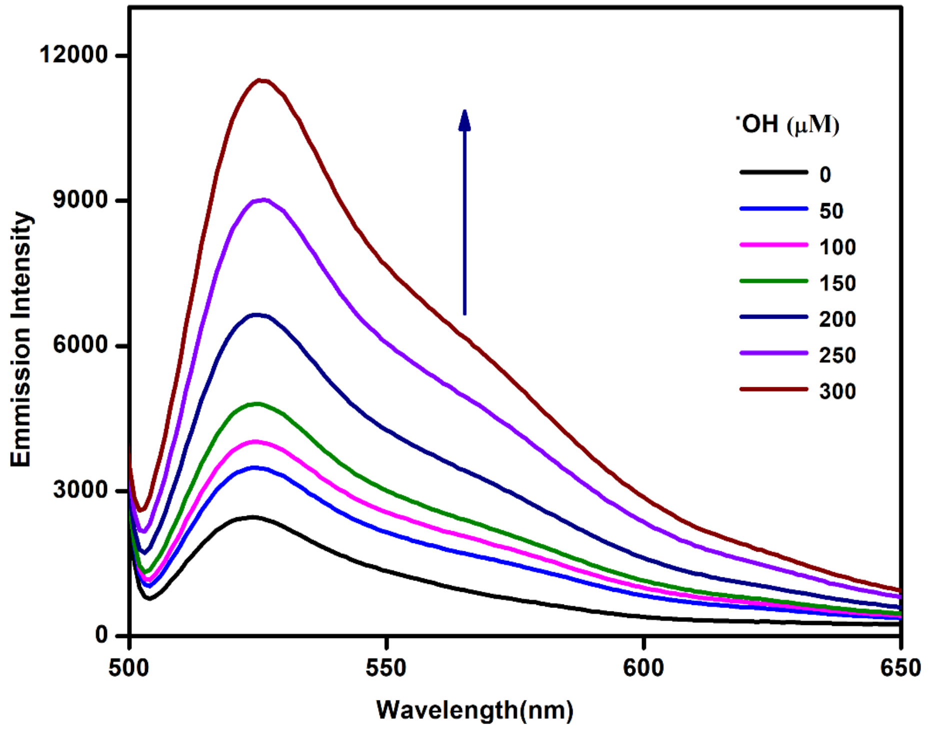

Figure S18. Emission of **9** (10 µM, methanol/water: v/v = 70:30) with increasing concentrations of ˙OH (0–300 µM); (λ_{ex} = 485 nm and λ_{em} = 525 nm).

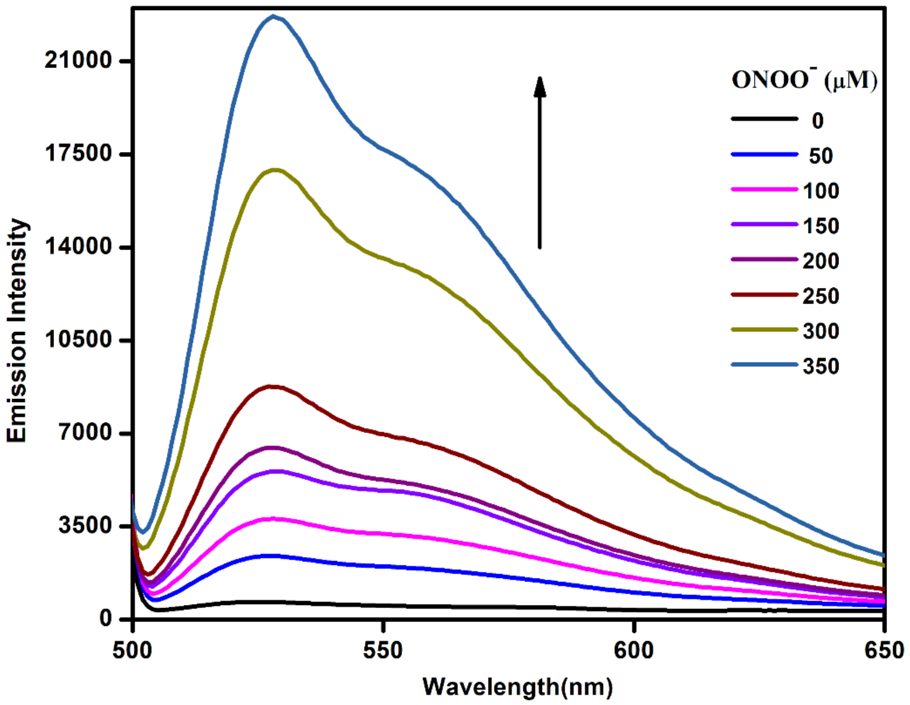

Figure S19. Emission of **9** (10 µM, methanol/water: v/v = 70:30) with increasing concentrations of $ONOO^-$ (0–300 µM); (λ_{ex} = 485 nm and λ_{em} = 525 nm).

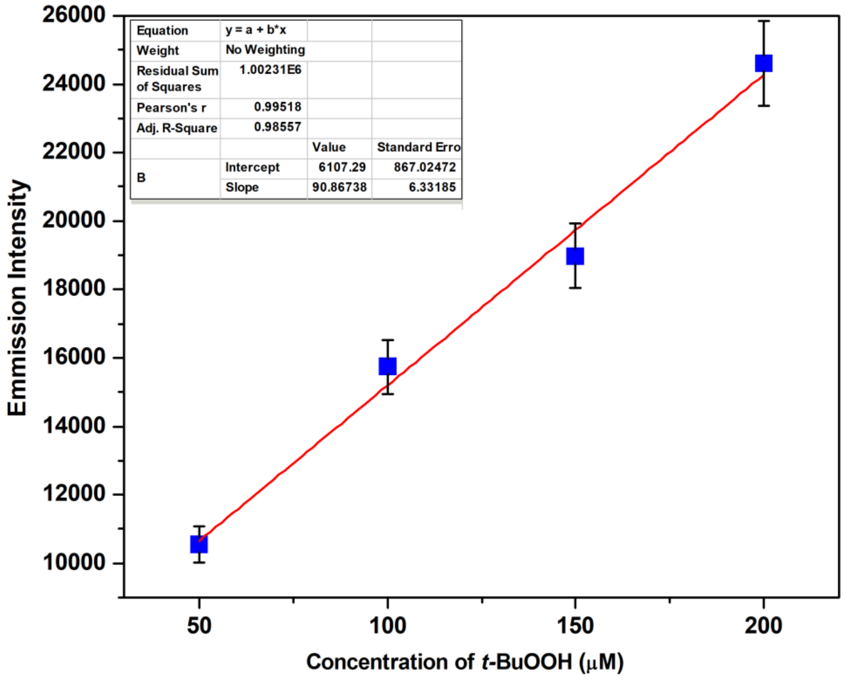

Figure S20. Plot for the calculation of LOD from the emission of **9** (10 µM, methanol/water: v/v = 70:30) with increasing concentrations of *t*-BuOOH (0–300 µM); (λ_{ex} = 485 nm and λ_{em} = 525 nm).

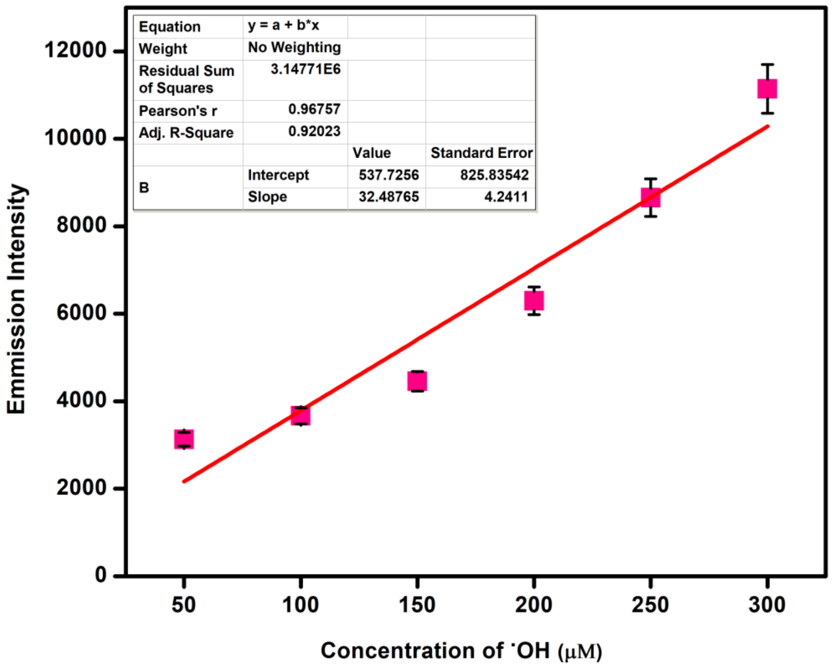

Figure S21. Plot for the calculation of LOD from the emission of **9** (10 μM, methanol/water: v/v = 70:30) with increasing concentrations of ˙OH (0–300 μM); (λ_{ex} = 485 nm and λ_{em} = 525 nm).

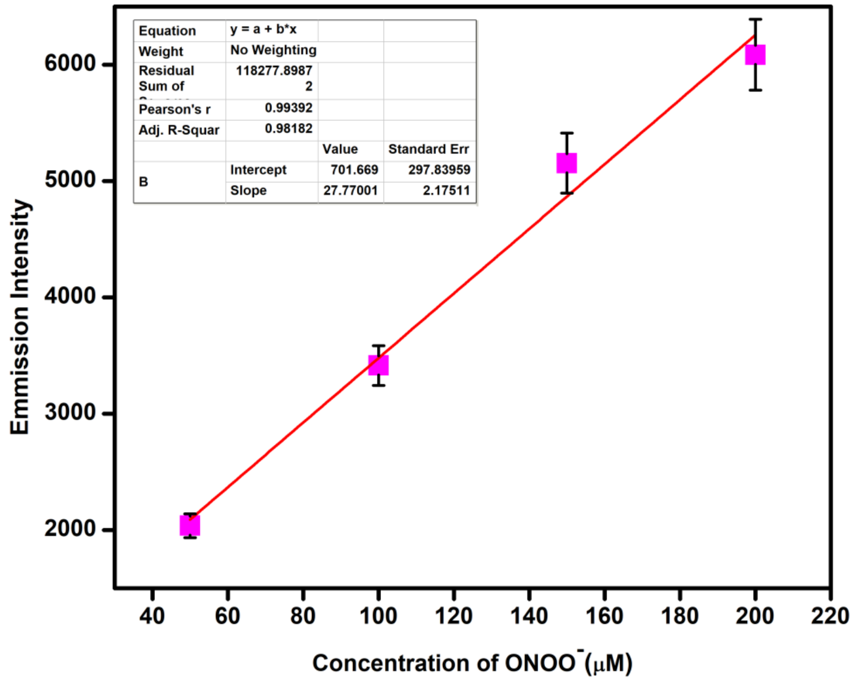

Figure S22. Plot for the calculation of LOD from the emission of **9** (10 μM, methanol/water: v/v = 70:30) with increasing concentrations of $ONOO^-$ (0–200 μM); (λ_{ex} = 485 nm and λ_{em} = 525 nm).

Table S8. Optimized geometries and Cartesian coordinates of probe **9** and its selenoxide **III** on the B3LYP/6-311+G(d,p) level

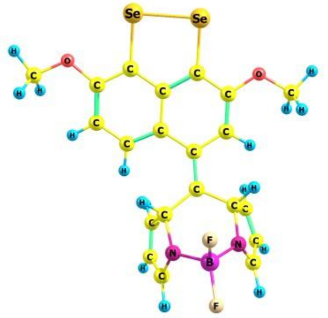

Probe **9**
Sum of Electronic and Thermal Enthalpies = -6098.072687 a.u.

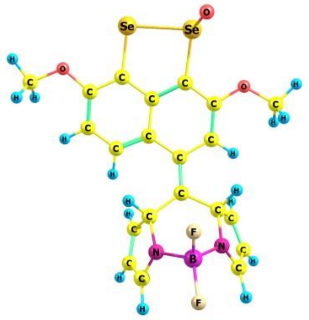

Selenoxide **III**
Sum of Electronic and Thermal Enthalpies = -6173.263014 a.u.

Probe 9				Selenoxide III			
6	-2.753793000	1.390061000	-0.064156000	6	-2.865090000	1.400348000	-0.070343000
1	-2.053184000	1.969688000	0.552948000	1	-2.120468000	1.941187000	0.535421000
6	-3.046651000	2.094050000	-1.364715000	6	-3.286270000	2.278243000	-1.220783000
1	-2.324218000	2.208331000	-2.157825000	1	-2.646549000	2.503050000	-2.060574000
6	-4.332982000	2.547765000	-1.351208000	6	-4.542918000	2.742997000	-1.003543000
1	-4.851436000	3.088531000	-2.129028000	1	-5.128030000	3.402217000	-1.627255000
6	-4.906996000	2.156861000	-0.109367000	6	-4.992944000	2.165959000	0.227669000
1	-5.917144000	2.330636000	0.236232000	1	-5.959348000	2.289000000	0.699740000
7	-4.072023000	1.437599000	0.619570000	7	-4.107051000	1.352138000	0.743600000
6	-2.200617000	-0.013509000	-0.243561000	6	-2.296600000	0.035649000	-0.409272000
6	-3.178183000	-1.183770000	-0.167392000	6	-3.225278000	-1.170613000	-0.337800000
1	-2.696790000	-1.987654000	0.419546000	1	-2.658922000	-1.985966000	0.156502000
6	-3.610071000	-1.682017000	-1.513610000	6	-3.771841000	-1.677194000	-1.636917000
6	-5.451767000	-1.258329000	-0.296835000	6	-5.483159000	-1.408969000	-0.210220000
1	-2.916676000	-1.961126000	-2.292450000	1	-3.149283000	-1.901095000	-2.490570000
6	-4.976250000	-1.731976000	-1.549844000	6	-5.119822000	-1.837038000	-1.524787000
1	-6.478087000	-1.142886000	0.023757000	1	-6.472719000	-1.377557000	0.226877000
1	-5.604871000	-2.045783000	-2.370019000	1	-5.813218000	-2.195907000	-2.270695000
7	-4.459170000	-0.877468000	0.493385000	7	-4.445602000	-0.964305000	0.461960000
5	-4.525681000	0.263301000	1.556568000	5	-4.459614000	0.089291000	1.623673000
9	-3.633179000	0.057572000	2.583652000	9	-3.500329000	-0.165458000	2.564436000
9	-5.839104000	0.464844000	1.957992000	9	-5.748330000	0.223187000	2.116331000
6	-0.833549000	-0.319086000	-0.398267000	6	-0.929228000	-0.225431000	-0.609767000
6	0.284844000	0.644721000	-0.364443000	6	0.163684000	0.762953000	-0.518757000
6	-0.461629000	-1.703298000	-0.478141000	6	-0.524151000	-1.588510000	-0.812157000
6	0.186596000	2.033151000	-0.585811000	6	0.022614000	2.155825000	-0.680012000
6	1.616125000	0.144432000	-0.120059000	6	1.501583000	0.285094000	-0.281146000
6	0.839095000	-2.148568000	-0.283706000	6	0.786343000	-2.012749000	-0.608684000
1	-1.211002000	-2.447468000	-0.680998000	1	-1.240904000	-2.326502000	-1.127469000
6	1.266581000	2.899542000	-0.494632000	6	1.074124000	3.046272000	-0.527728000
1	-0.748663000	2.478728000	-0.870780000	1	-0.917830000	2.582508000	-0.978851000
6	2.701884000	1.044961000	-0.003257000	6	2.560895000	1.205980000	-0.103523000
6	1.867593000	-1.244332000	-0.052265000	6	1.781566000	-1.102816000	-0.274320000
6	2.538589000	2.409393000	-0.173260000	6	2.354584000	2.573120000	-0.204191000
1	1.111169000	3.954071000	-0.678608000	1	0.896194000	4.102859000	-0.675020000
34	4.460854000	0.363891000	0.347912000	34	4.336121000	0.529501000	0.161352000
34	3.638016000	-1.883939000	0.255433000	34	3.513339000	-1.794351000	0.279183000
8	1.217337000	-3.463263000	-0.289573000	8	1.182645000	-3.312639000	-0.721614000
8	3.664569000	3.169291000	-0.036969000	8	3.448252000	3.367343000	-0.025256000
6	0.235179000	-4.473594000	-0.477550000	6	0.209293000	-4.346624000	-0.790037000
1	-0.237253000	-4.392846000	-1.462071000	1	-0.347251000	-4.313117000	-1.732881000
1	0.769545000	-5.419919000	-0.412991000	1	0.767990000	-5.279715000	-0.740248000
1	-0.530007000	-4.434469000	0.304535000	1	-0.484009000	-4.291507000	0.054770000
6	3.574124000	4.575014000	-0.237638000	6	3.297881000	4.778601000	-0.106278000
1	3.251454000	4.810467000	-1.257042000	1	2.977039000	5.089732000	-1.106185000
1	2.889685000	5.035522000	0.482085000	1	2.586686000	5.147012000	0.640383000
1	4.579205000	4.961592000	-0.078692000	1	4.283626000	5.192116000	0.099771000
				8	3.326395000	-2.257312000	1.865556000

Figure S23. NBO charge density diagram of probe **9** at B3LYP/6-311++G(d,p) level on the B3LYP/6-311+G(d,p) level-optimized geometry.

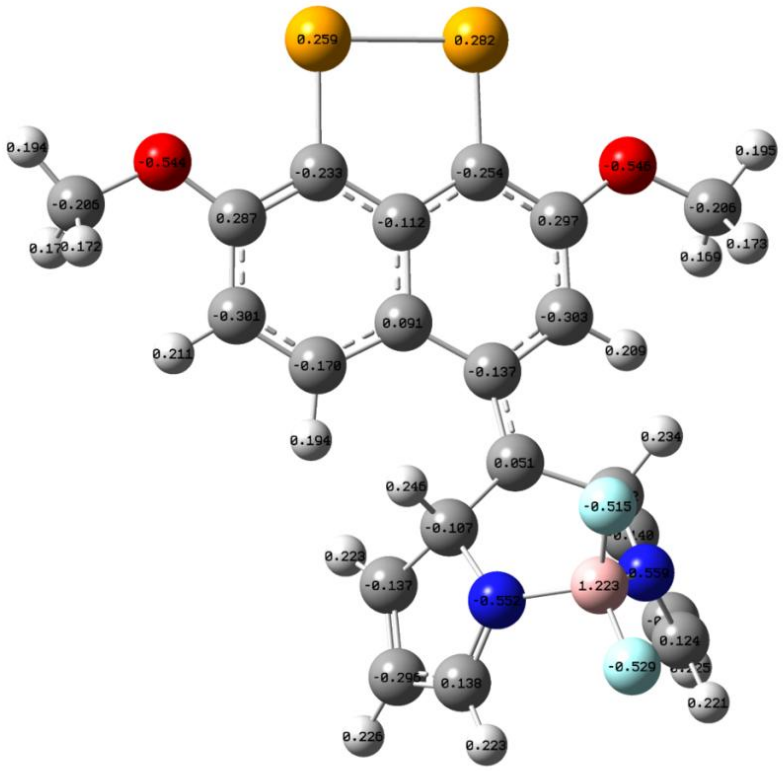

Figure S24. Expansion of mass spectrum of mixture of probe **9** and H_2O_2

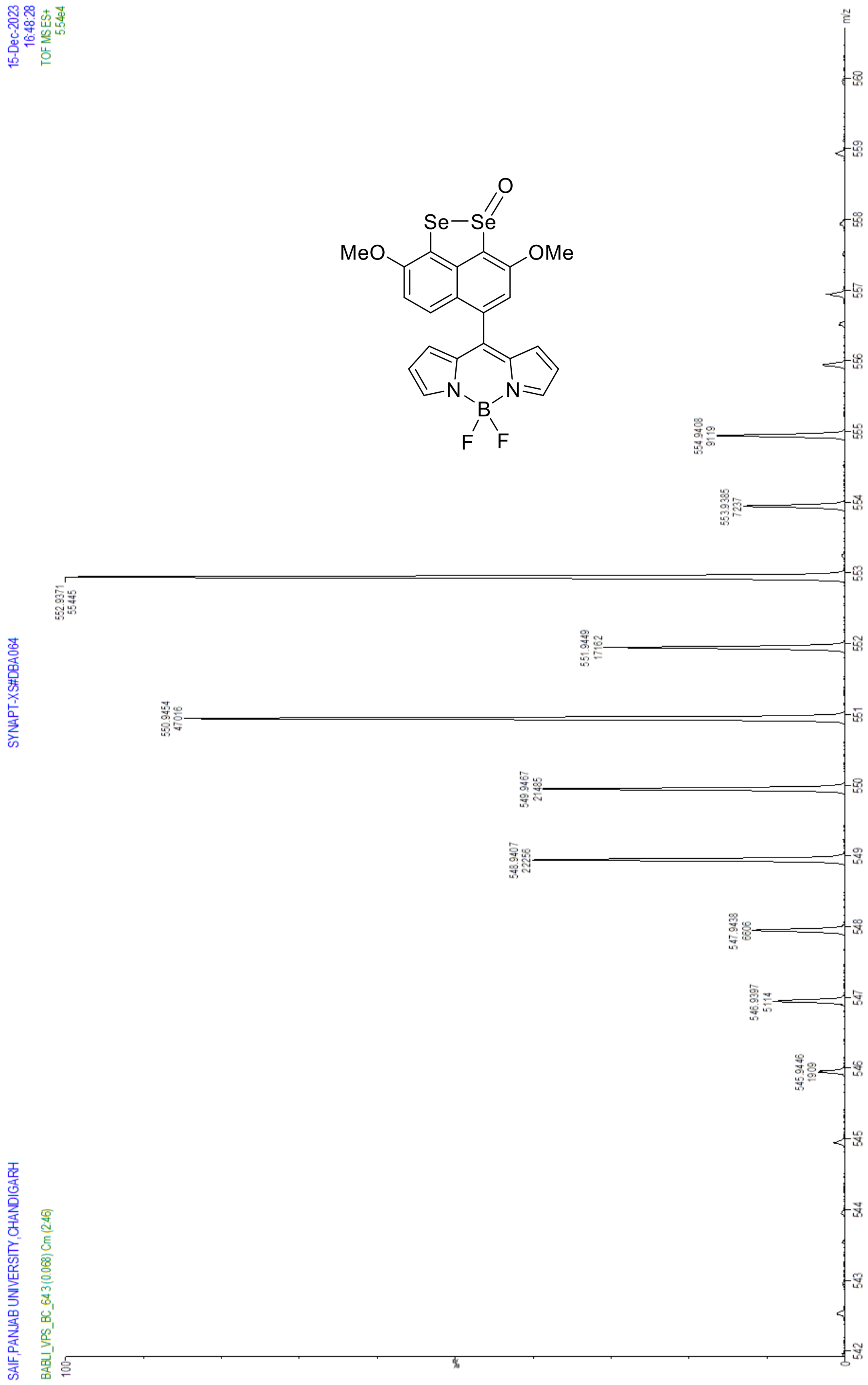

Figure S25. HOMO-LUMO diagram of probe **9** using the B3LYP/6-311+G(d,p) level-optimized geometry.

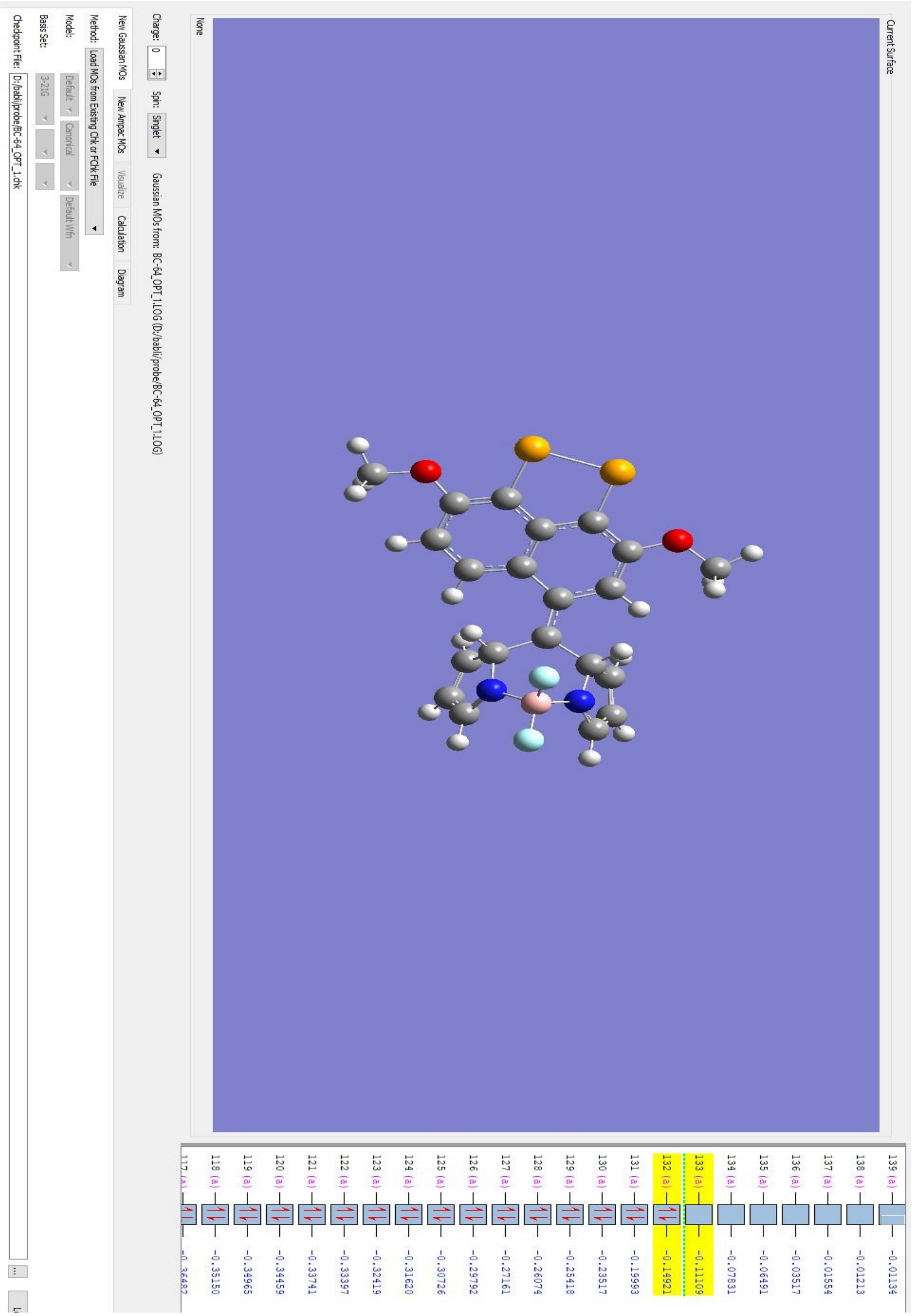

Figure S26. HOMO-LUMO diagram of selenoxide **III** using the B3LYP/6-311+G(d,p) level-optimized geometry.

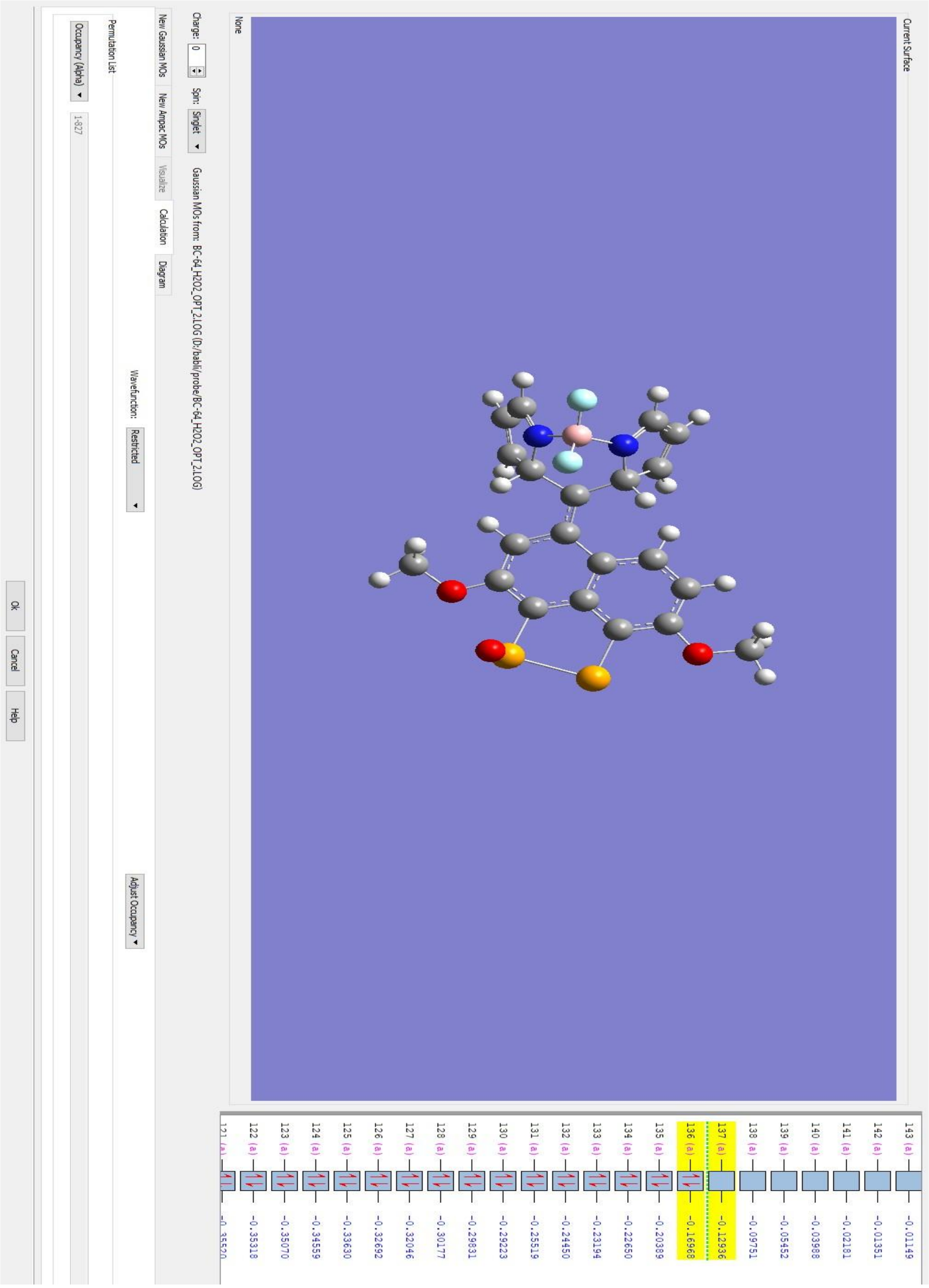

Supplement: Supplementary file 1 — ao4c05366_si_001.pdf [file ao4c05366_si_001.pdf]
